# Pooling analysis regarding the impact of human vitamin D receptor variants on the odds of psoriasis

**DOI:** 10.1186/s12881-019-0896-6

**Published:** 2019-10-17

**Authors:** Juan Li, Li Sun, Jinghui Sun, Min Yan

**Affiliations:** Department of Dermatology, Shengli Oilfield Central Hospital, Jinan Road no. 31, 257000 Dongying, Shandong People’s Republic of China

**Keywords:** *VDR*, Psoriasis, Polymorphism, Meta-analysis

## Abstract

**Background:**

The study aims at scientifically investigating the genetic effect of four polymorphisms (*rs7975232*, *rs1544410*, *rs2228570*, and *rs731236*) within the human Vitamin D Receptor (*VDR*) gene on the odds of psoriasis through an updated meta-analysis.

**Methods:**

We searched eight databases and screened the studies for pooling. Finally, a total of eighteen eligible case-control studies were included. BH (Benjamini & Hochberg) adjusted *P-*values of association (*P*_association_) and odd ratios (ORs) with the corresponding 95% confidence intervals (CIs) were calculated under the allele, homozygote, heterozygote, dominant, recessive, and carrier models.

**Results:**

Compared with the negative controls, no statistically significant difference in the odds of psoriasis was detected for the cases under any genetic models (BH adjusted *P*_*association*_ > 0.05). We also performed subgroup meta-analyses by the source of controls, ethnicity, country, Hardy-Weinberg equilibrium, and genotyping method. Similar results were observed in most subgroup meta-analyses (BH adjusted *P*_*association*_ > 0.05). Besides, data of Begg’s and Egger’s tests excluded the significant publication bias; while the sensitivity analysis data further indicated the statistical reliability of our pooling results.

**Conclusion:**

The currently available data fails to support a robust association between *VDR rs7975232*, *rs1544410*, *rs2228570* and *rs731236* polymorphisms and psoriasis susceptibility, which still required the support of more case-control studies.

## Background

Vitamin D Receptor (VDR) protein, a member of the nuclear receptor superfamily of ligand-activated transcription factors, is thought to be implicated in several cell biological events (e.g., calcium and phosphate homeostasis, cell differentiation and apoptosis) [1, 2]. The human *VDR* gene is mapped on chromosome 12 and contains four common polymorphisms, namely *rs7975232* A/C in intron eight (A*pa*I) *rs1544410* G/A in intron eight (B*sm*I), *rs2228570* T/C in exon two (F*ok*I), and *rs731236* T/C in exon nine (TaqI) [3–5]. In addition, linkage disequilibrium exists among the *rs7975232*, *rs1544410,* and *rs731236* polymorphisms [6, 7]. Here, we investigated the possible role of *VDR rs7975232*, *rs1544410*, *rs2228570*, and *rs731236* polymorphisms in the susceptibility to psoriasis disease.

Psoriasis is a type of chronic inflammatory immune-mediated disease with discrete, erythematous scaly plaques on the skin, and is characterized by the abnormal proliferation of keratinocytes and disordered maturation of the epidermis [8–10]. Genetic factors are potentially linked to the occurrence or pathogenesis of psoriasis [11, 12]. We observed the open questions of the association between the *VDR* polymorphisms and psoriasis susceptibility among different populations. For instance, the *rs7975232* polymorphism of *VDR* was reportedly associated with the psoriasis risks in the Korean population [13, 14], Chinese population [15], or Turkish population [16, 17]. However, the *VDR rs7975232* polymorphism was not considered a risk factor for psoriasis cases in Japan [18], Italy [19], Croatia [20], or Egypt [21]. Therefore, it is meaningful to conduct a meta-analysis to pool the relevant data for a comprehensive assessment of this issue. Even though a recent meta-analysis was conducted by searching three databases in February 2018 [3], the publication of possible new data, different database retrieval, data collection and analysis strategies led us to perform another updated comprehensive pooling analysis and a series of followed stratification analysis, of gene-disease association up to August 18, 2019.

## Methods

### Database retrieval

Referring to the HuGENet™ HuGE Review Handbook, version 1.0, we retrieved the relevant publications from eight online databases, including PubMed, Web of Science (WOS), Excerpta Medica Database (EMBASE), China National Knowledge Infrastructure (CNKI), WANFANG, OVID, Scopus and Cochrane, up to August 18, 2019, without any restrictions regarding geographical, language or publication time. We provided the searching terms in Additional file 1: Table S1.

### Inclusion and exclusion criteria

Three investigators (J. Li, L. Sun, and J. Sun) designed the inclusion and exclusion criteria, independently screened the above articles, and evaluated the eligibility. Inclusion criteria: (1) comparing psoriasis cases versus negative controls; (2) detecting the *VDR* polymorphisms; (3) containing the major/minor allele frequency or completed genotype distribution. Exclusion criteria: (1) non-human studies; (2) reviews; (3) meeting or conference abstracts; (4) meta-analyses; (5) other diseases; (6) other genes; (7) expression or non-single nucleotide polymorphism (SNP); (8) duplicate or overlapped data.

### Data collecting

Two investigators (J. Li and L. Sun) designed a form and independently collected the information, including the first author, publication year, ethnicity, source of controls, gender, age, calcipotriol response, family history, genotyping method and genotype frequency. Based on the genotype frequency distribution, we utilized the chi-square test to calculate the *P-*value of HWE. The summarized data were assessed together for errors. When the frequency data were missing, the investigator (M. Yan) sent an email to the corresponding author. In addition, two investigators (J. Li and L. Sun) assessed the study quality using the Newcastle-Ottawa quality assessment scale (NOS) where scores range between 1 and 9. When a disagreement was encountered, we discussed with the third investigator (M. Yan) to obtain consensus. We considered studies high quality when the NOS score ≥ 5.

### Tests for association, heterogeneity

After data sorting via Microsoft Excel 2016, STATA 12.0 software (StataCorp, USA) was applied to obtain the *P*-value of association, ORs and 95% CI under the allele (allele C vs. A for *VDR rs7975232* polymorphism; allele A vs. G for *rs1544410* polymorphism; allele C vs. T for *rs2228570* polymorphism; allele C vs. T for *rs731236* polymorphism), homozygote (CC vs. AA; AA vs. GG; CC vs. TT; CC vs. TT), heterozygote (AC vs. AA; GA vs. GG; TC vs. CC; TC vs. TT), dominant (AC + CC vs AA; GA + AA vs. GG; TC + CC vs. TT; TC + CC vs. TT), recessive (CC vs. AA+AC; AA vs. GG + GA; CC vs. TT + TC; CC vs. TT + TC) and carrier (carrier C vs. A; carrier A vs. G; carrier C vs. T; carrier C vs. T) models. *We utilized the BH (Benjamini & Hochberg) correction method to adjust the P*_*association*_ *value through the p.adjust () function of R software version 3.4.4. BH-corrected P*_*association*_ < 0.05 from the association test was considered statistically significant.

>Based on the “meta-analysis of binary data” function of STATA 12.0 software, we obtained the I^2^ value (variation in ORs attributable to heterogeneity) and *P*-value of heterogeneity. When *P*-value < 0.05 or the I^2^ value > 50%, we utilized the random-effect pooling model (DerSimonian and Laird method); Otherwise, we used a fixed-effect model (Mantel-Haenszel method). To assess data stability and the source of potential heterogeneity, we conducted a series of subgroup analyses based on the factors of the control source, ethnicity, country, HWE, and genotyping method.

We performed the sensitivity analyses under all the genetic models, through the “influence analysis, metan-based (metaninf)” function of STATA 12.0 software. Upon the exclusion of each study one by one, the lack of largely affected meta-analysis estimates in figures suggested the statistical stability of data. If not, the omitted studies are deemed as the source of heterogeneity.

### Tests for publication bias

We also performed the Begg’s test and Egger’s test to evaluate the potential publication bias through the “Publication Bias (metabias)” function of STATA 12.0 software. Begg’s funnel plot and Egger’s publication bias plot were generated, respectively. The basically symmetrical funnel plot, *P*-values for Begg’s test and Egger’s test greater than 0.05 indicate the absence of larger publication bias.

## Results

### Case-control study identification

**Fig. 1:**
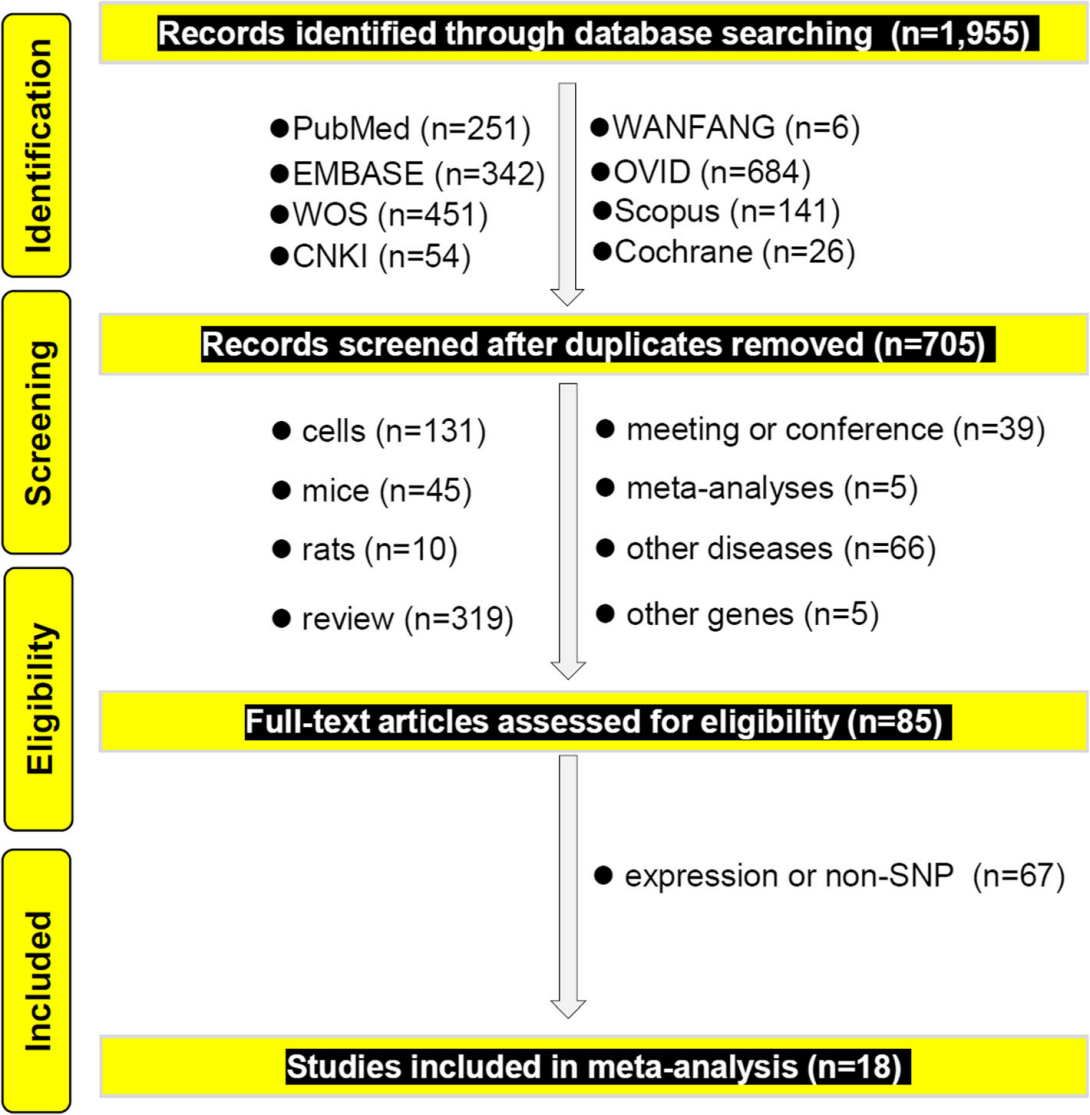
The flow chart of the eligible case-control study identification

Figure 1 presents the flow chart of study identification. We first retrieved 1955 records from eight on-line databases [PubMed (*n* = 251), EMBASE (*n* = 342), WOS (*n* = 451), CNKI (*n* = 54), WANFANG (*n* = 6), OVID (*n* = 684), Scopus (*n* = 141) and Cochrane (*n* = 26)]. We then screened a total of 705 records after removing duplicate records from different databases. Next, we excluded an additional 620 records per the exclusion criteria. The detailed information was shown in Fig. 1. After assessing the eligibility of 85 full-text articles, we removed an additional 67 articles with “expression or non-SNP” data. Finally, we included a total of 18 case-control studies [13–30] for our meta-analysis. We also summarized and listed the genotypic distribution (Table 1) and clinical characteristics, (Additional file 2: Table S2). No low-quality studies with a NOS quality score ≥ five were included in this analysis (Additional file 3: Table S3).

**Table 1:** Genotype distributions of included case-control studies

First author	Year	Ethnicity	case			polymorphism	Control			Source of controls	*P*_HWE_	Genotyping method
			XX	XY	YY		XX	XY	YY			
Dayangac	2007	Caucasian	12	29	10	*rs7975232*	30	55	15	PB	0.21	PCR-RFLP
Kaya	2002	Caucasian	14	31	8	*rs7975232*	27	21	6	PB	0.54	PCR-RFLP
Lee	2002	Asian	5	28	22	*rs7975232*	3	29	72	PB	0.97	PCR-RFLP
Liu	2017	Asian	39	56	15	*rs7975232*	100	67	16	PB	0.33	LDR
Okita	2002	Asian	4	19	27	*rs7975232*	9	41	36	PB	0.59	PCR-RFLP
Park	1999	Asian	10	46	48	*rs7975232*	3	29	72	PB	0.97	PCR-RFLP
Richetta	2014	Caucasian	37	50	21	*rs7975232*	88	136	44	PB	0.48	Taqman assay
Rucevic	2012	Caucasian	48	99	33	*rs7975232*	110	193	63	PB	0.17	PCR-RFLP
Saeki	2002	Asian	9	46	60	*rs7975232*	10	26	33	PB	0.21	PCR-RFLP
Zhao	2015	Asian	159	148	17	*rs7975232*	92	54	12	PB	0.31	gene sequencing
Zhou	2014	Asian	182	130	30	*rs7975232*	209	113	19	HB	0.47	Multiplex SNapSHOT
Zhu	2002	Asian	22	30	60	*rs7975232*	8	48	52	PB	0.49	PCR-RFLP
Zuel	2011	African	23	24	3	*rs7975232*	18	30	2	PB	0.02	PCR-RFLP
Kaya	2002	Caucasian	10	25	18	*rs1544410*	11	22	21	PB	0.25	PCR-RFLP
Kontula	1997	Caucasian	2	12	5	*rs1544410*	10	29	36	PB	0.29	PCR-RFLP
Lee	2002	Asian	1	3	51	*rs1544410*	0	13	88	PB	0.49	PCR-RFLP
Liu	2017	Asian	97	11	2	*rs1544410*	163	18	2	PB	0.08	LDR
Mee	1998	Caucasian	78^a^	106^a^		*rs1544410*	114^a^	134^a^		NA	> 0.05	PCR-RFLP
Okita	2002	Asian	3	7	40	*rs1544410*	4	12	70	PB	0.00	PCR-RFLP
Richetta	2014	Caucasian	42	42	24	*rs1544410*	87	124	57	PB	0.30	Taqman assay
Rucevic	2012	Caucasian	19	94	67	*rs1544410*	68	175	123	PB	0.68	PCR-RFLP
Ruggiero	2004	Caucasian	17	28	15	*rs1544410*	18	32	18	PB	0.63	PCR-RFLP
Saeki	2002	Asian	4	12	99	*rs1544410*	8	10	51	PB	0.00	PCR-RFLP
Zhao	2015	Asian	306	17	1	*rs1544410*	142	16	0	PB	0.50	gene sequencing
Zhou	2014	Asian	311	29	2	*rs1544410*	321	19	1	HB	0.22	Multiplex SNapSHOT
Zhu	2002	Asian	0	36	76	*rs1544410*	0	16	92	PB	0.41	PCR-RFLP
Dayangac	2007	Caucasian	28	20	3	*rs2228570*	55	36	9	PB	0.39	PCR-RFLP
Halsall	2005	Caucasian	250^a^	160^a^		*rs2228570*	102^a^	58^a^		HB	> 0.05	PCR-RFLP
Kaya	2002	Caucasian	24	23	6	*rs2228570*	29	22	3	PB	0.66	PCR-RFLP
Liu	2017	Asian	25	61	24	*rs2228570*	50	97	36	PB	0.37	LDR
Richetta	2014	Caucasian	41	49	18	*rs2228570*	117	114	37	PB	0.28	Taqman assay
Saeki	2002	Asian	37	55	23	*rs2228570*	29	31	9	PB	0.87	PCR-RFLP
Zhao	2015	Asian	118	150	56	*rs2228570*	25	68	65	PB	0.31	gene sequencing
Zhou	2014	Asian	94	180	68	*rs2228570*	99	171	71	HB	0.86	Multiplex SNapSHOT
Acikbas	2012	Caucasian	14	47	41	*rs731236*	27	33	42	PB	< 0.05	PCR-RFLP
Dayangac	2007	Caucasian	26	23	2	*rs731236*	35	49	16	PB	0.87	PCR-RFLP
Halsall	2005	Caucasian	262^a^	148^a^		*rs731236*	90^a^	70^a^		HB	> 0.05	PCR-RFLP
Kaya	2002	Caucasian	19	25	9	*rs731236*	22	24	8	PB	0.73	PCR-RFLP
Liu	2017	Asian	101	9	0	*rs731236*	171	12	0	PB	0.65	LDR
Okita	2002	Asian	39	11	0	*rs731236*	72	14	0	PB	0.41	PCR-RFLP
Richetta	2014	Caucasian	42	44	22	*rs731236*	89	131	48	PB	0.99	Taqman assay
Rucevic	2012	Caucasian	79	82	19	*rs731236*	139	175	52	PB	0.80	PCR-RFLP
Saeki	2002	Asian	100	14	1	*rs731236*	51	16	2	PB	0.59	PCR-RFLP
Zhao	2015	Asian	283	37	4	*rs731236*	129	27	2	PB	0.67	gene sequencing
Zhou	2014	Asian	308	33	1	*rs731236*	315	26	0	HB	0.46	Multiplex SNapSHOT
Zuel	2011	African	16	25	9	*rs731236*	19	26	5	PB	0.36	PCR-RFLP

*X* major allele, *Y* minor allele, *PB* population-based controls, *HB* hospital-based controls, *NA* not available data, *PCR-RFLP* polymerase chain reaction-restriction fragment length polymorphism, *P*_HWE_ *P*-value of Hardy-Weinberg equilibrium, *LDR* ligase detection reactions

^a^ The frequency of major allele and minor allele

### *VDR rs7975232* polymorphism

**Fig. 2:**
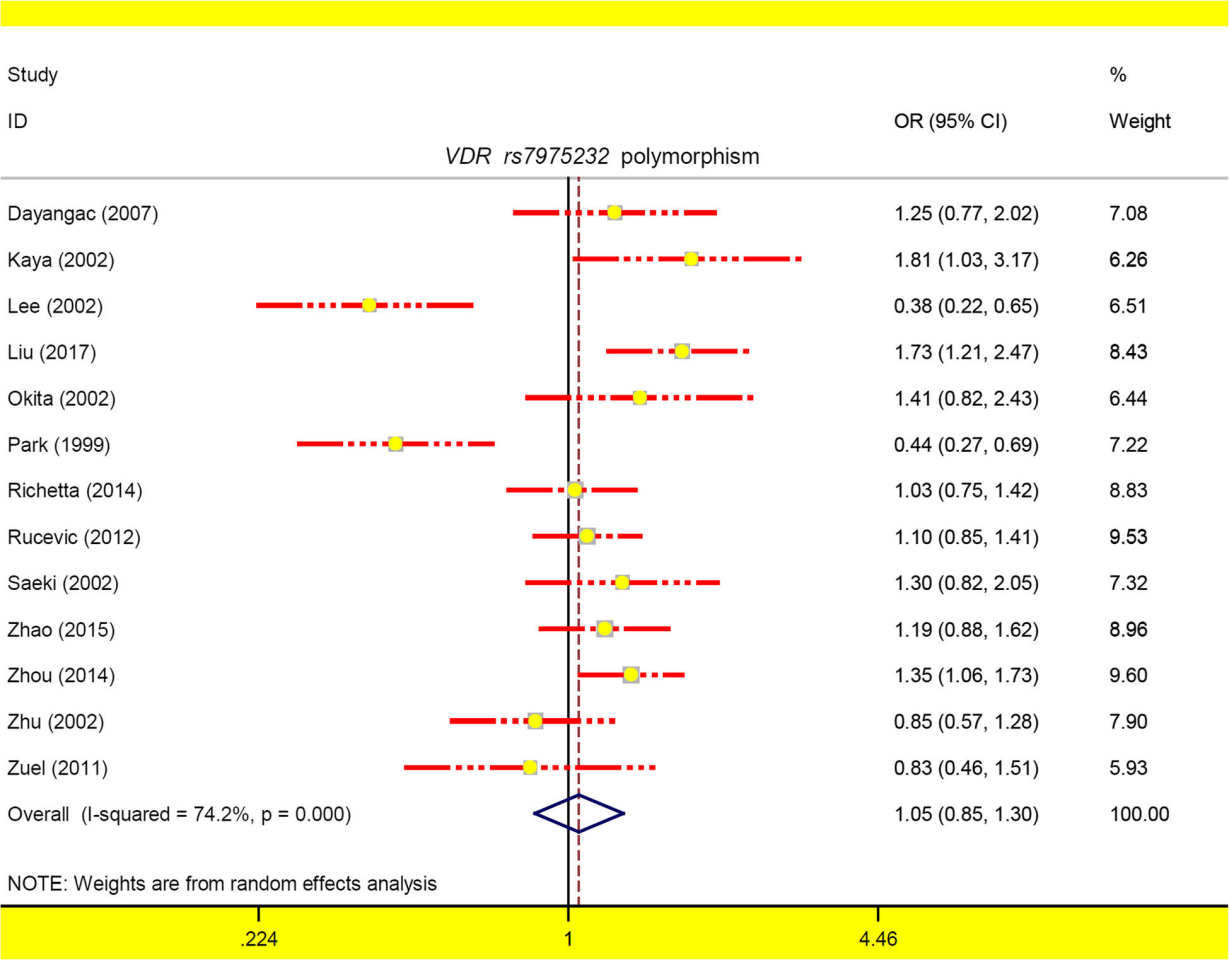
The forest plot for *VDR rs7975232* polymorphism under the allele model

There are a total of thirteen case-control studies with 1654 cases and 1991 controls for the meta-analysis of the *VDR rs7975232* polymorphism and psoriasis susceptibility. The heterogeneity under the carrier C vs. A model (Table 2, I^2^ = 42.3%, *P*_heterogeneity_ = 0.053) led to the utilization of a random-effects pooling model, and a fixed-effects pooling model was utilized for the other genetic models. Pooling results of Table 2 showed no statistically significant difference in the odds of psoriasis between cases and controls under the following six genetic models: allele C vs. A [*P*_*association*_ (*P*-value of association) =0.640, BH-adjusted *P*_*association*_ = 0.960], homozygote CC vs. AA (*P*_*association*_ = 0.585, BH-adjusted *P*_*association*_ = 0.960), heterozygote AC vs. AA (*P*_*association*_ = 0.370, BH-adjusted *P*_*association*_ = 0.960), dominant AC + CC vs. AA (*P*_*association*_ = 0.356, BH-adjusted *P*_*association*_ = 0.960), recessive CC vs. AA+AC (*P*_*association*_ = 0.928, BH-adjusted *P*_*association*_ = 0.977), and carrier C vs. A (*P*_*association*_ = 0.977, BH-adjusted *P*_*association*_ = 0.977). Figure 2 presents the forest plot under the allele model.

**Table 2:** Pooled analyses of the association between *VDR rs7975232* polymorphism and susceptibility to psoriasis

Models	M	I^2^	*P*_heterogeneity_	Stratification	case/control (N)	OR [95% CI]	*P*_*association*_	BH
allele C vs. A	R	74.2%	< 0.001	overall	1654/1991 (13)	1.05 [0.85~1.30]	0.640	0.960
		83.4%	< 0.001	Asian	1212/1153 (8)	0.980 [0.70~1.38]	0.921	0.921
		6.4%	0.361	Caucasian	392/788 (4)	1.16 [0.96~1.39]	0.123	0.346
		74.6%	< 0.001	PB	1312/1650 (12)	1.02 [0.81~1.30]	0.849	0.856
		57.4%	0.071	China	888/790 (4)	1.26 [0.99~1.61]	0.065	0.195
		75.8%	< 0.001	*P*_HWE_ > 0.05	1604/1941 (12)	1.07 [0.85~1.33]	0.567	0.740
		76.2%	< 0.001	PCR-RFLP	770/1041 (9)	0.93 [0.69~1.27]	0.668	0.819
CC vs. AA	R	55.6%	0.008	overall	1654/1991 (13)	1.11 [0.76~1.64]	0.585	0.960
		71.9%	0.001	Asian	1212/1153 (8)	0.91 [0.48~1.71]	0.761	0.921
		0.0%	0.653	Caucasian	392/788 (4)	1.31 [0.91~1.90]	0.147	0.346
		55.7%	0.010	PB	1312/1650 (12)	1.04 [0.69~1.59]	0.838	0.856
		72.6%	0.012	China	888/790 (4)	1.11 [0.76~1.64]	0.718	0.718
		59.3%	0.005	*P*_HWE_ > 0.05	1604/1941 (12)	1.11 [0.74~1.65]	0.617	0.740
		60.5%	0.009	PCR-RFLP	770/1041 (9)	0.93 [0.52~1.66]	0.803	0.819
AC vs. AA	R	61.0%	0.002	overall	1654/1991 (13)	1.15 [0.85~1.54]	0.370	0.960
		68.2%	0.003	Asian	1212/1153 (8)	1.10 [0.70~1.72]	0.683	0.921
		45.9%	0.136	Caucasian	392/788 (4)	1.27 [0.84~1.91]	0.257	0.346
		64.0%	0.001	PB	1312/1650 (12)	1.11 [0.78~1.57]	0.578	0.856
		83.2%	< 0.001	China	888/790 (4)	1.15 [0.64~2.07]	0.638	0.718
		60.6%	0.003	*P*_HWE_ > 0.05	1604/1941 (12)	1.20 [0.89~1.63]	0.235	0.478
		62.2%	0.007	PCR-RFLP	770/1041 (9)	0.94 [0.58~1.54]	0.819	0.819
AC + CC vs. AA	R	63.5%	0.001	overall	1654/1991 (13)	1.15 [0.86~1.54]	0.356	0.960
		71.6%	0.001	Asian	1212/1153 (8)	1.06 [0.68~1.66]	0.800	0.921
		43.5%	0.151	Caucasian	392/788 (4)	1.30 [0.89~1.90]	0.179	0.346
		66.0%	0.001	PB	1312/1650 (12)	1.10 [0.78~1.55]	0.595	0.856
		79.1%	0.002	China	888/790 (4)	1.24 [0.75~2.04]	0.402	0.603
		63.6%	0.001	*P*_HWE_ > 0.05	1604/1941 (12)	1.20 [0.89~1.62]	0.239	0.478
		65.8%	0.003	PCR-RFLP	770/1041 (9)	0.93 [0.57~1.52]	0.771	0.819
CC vs. AA+AC	R	63.9%	0.001	overall	1654/1991 (13)	1.01 [0.74~1.39]	0.928	0.977
		77.4%	< 0.001	Asian	1212/1153 (8)	0.91 [0.57~1.47]	0.712	0.921
		0.0%	0.943	Caucasian	392/788 (4)	1.19 [0.86~1.64]	0.295	0.346
		63.8%	0.001	PB	1312/1650 (12)	0.97 [0.69~1.35]	0.856	0.856
		20.8%	0.286	China	888/790 (4)	1.26 [0.88~2.14]	0.205	0.410
		66.7%	0.001	*P*_HWE_ > 0.05	1604/1941 (12)	1.00 [0.72~1.39]	0.977	0.977
		69.8%	0.001	PCR-RFLP	770/1041 (9)	0.93 [0.60~1.42]	0.727	0.819
carrier C vs. A	F	42.3%	0.053	overall	1654/1991 (13)	1.08 [0.96~1.21]	0.977	0.977
		63.1%	0.008	Asian	1212/1153 (8)	1.08 [0.93~1.25]	0.313	0.921
		0.0%	0.720	Caucasian	392/788 (4)	1.10 [0.90~1.34]	0.346	0.346
		43.6%	0.053	PB	1312/1650 (12)	1.04 [0.92~1.19]	0.507	0.856
		0.0%	0.578	China	888/790 (4)	1.23 [1.03~1.47]	0.020	0.120
		45.9%	0.041	*P*_HWE_ > 0.05	1604/1941 (12)	1.09 [0.96~1.22]	0.170	0.478
		46.3%	0.061	PCR-RFLP	770/1041 (9)	0.96 [0.82~1.13]	0.650	0.819

*M* statistical model, *R* random effect, *F* fixed effect, *P*_HWE_ *P*-value of Hardy-Weinberg equilibrium, *P*_heterogeneity_ *P*-value of Cochrane’s Q statistic for the assessment of heterogeneity, *N* Number of included case-control studies, *OR* odds ratio, *CI* confidence interval, *P*_*association*_ *P*-value of association

*BH* Benjamini & Hochberg-adjusted *P*_*association*_

We also performed subgroup meta-analyses based on the factors of control source, ethnicity, country, HWE, and genotyping method. We observed no significant differences between cases and controls in any subgroup (Table 2, all *P*_*association*_ > 0.05, BH-adjusted *P*_*association*_ > 0.05) except the subgroup of “China” under the carrier model (*P*_*association*_ = 0.020, BH-adjusted *P*_*association*_ = 0.120, OR = 1.23). Additional file 4: Figure S1 and Additional file 5: Figure S2 show the forest plots in the subgroup analysis by the factors of ethnicity and the source of controls (allele model). These results suggested that the *VDR rs7975232* polymorphism has no significant influence on the susceptibility to psoriasis.

### *VDR rs1544410* polymorphism

**Fig. 3:**
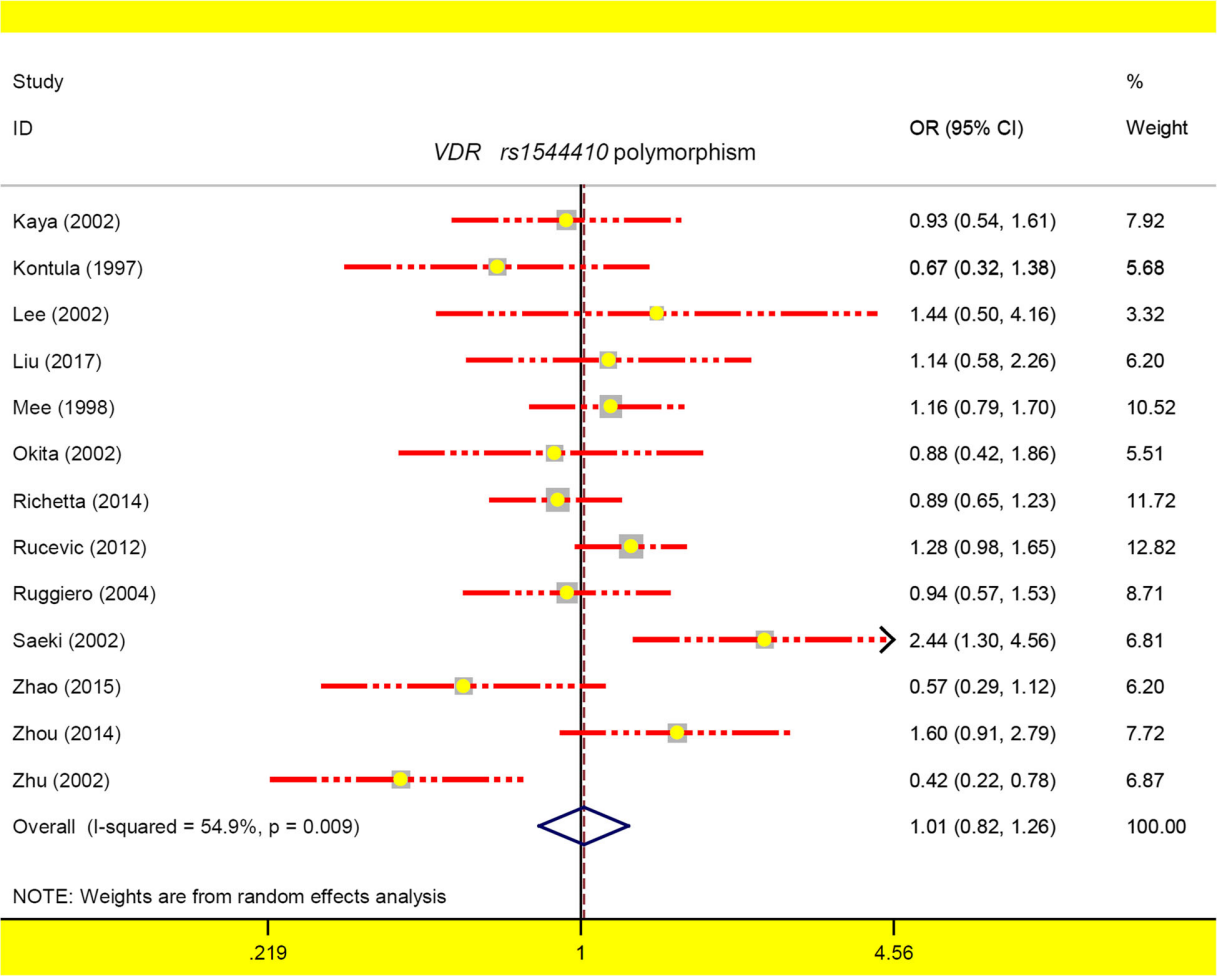
The forest plot for *VDR rs1544410* polymorphism under the allele model

For *VDR rs1544410,* thirteen studies containing 1620 cases/2001 controls were included. A random-effects pooling model was used for the allele A vs. G (Table 3, I^2^ = 54.9%, *P*_heterogeneity_ = 0.009), whereas a fixed-effects pooling model was utilized for the others (all I^2^ < 50.0%, *P*_heterogeneity_ > 0.05). We did not observe the statistical differences between cases and controls under any genetic model during the overall meta-analysis and subsequent subgroup analysis (Table 3, all *P*_*association*_ > 0.05, BH-adjusted *P*_*association*_ > 0.05) with the exception of the *“P*_HWE_ > 0.05” subgroup under the AA vs. GG + GA model (*P*_*association*_ = 0.018, BH-adjusted *P*_*association*_ = 0.108, OR = 0.99) and *“*PCR-RFLP” subgroup under the GG + GA vs. GG model (*P*_*association*_ = 0.035, BH-adjusted *P*_*association*_ = 0.144, OR = 1.46). Figure 3 presents a forest plot of the allele model in the overall meta-analysis, and Additional file 6: Figure S3 and Additional file 7: Figure S4 show the forest plots in the subgroup analysis by the factors of ethnicity and source of controls (allele model). These data suggested that the *VDR rs1544410* polymorphism seems not to be linked to the psoriasis susceptibility.

**Table 3:** Pooled analyses of the association between *VDR rs1544410* polymorphism and susceptibility to psoriasis

Models	M	I^2^	*P*_heterogeneity_	Stratification	case/control (N)	OR [95% CI]	*P*_*association*_	BH
allele A vs. G	R	54.9%	0.009	overall	1620/2001 (13)	1.01 [0.82~1.26]	0.898	0.925
		71.8%	0.002	Asian	1108/1046 (7)	1.04 [0.63~1.69]	0.889	0.973
		4.8%	0.386	Caucasian	512/955 (6)	1.05 [0.89~1.24]	0.547	0.821
		58.2%	0.008	PB	1186/1536 (11)	0.95 [0.74~1.23]	0.711	0.971
		75.0%	0.007	China	888/790 (4)	0.82 [0.43~1.54]	0.533	1.000
		61.8%	0.004	*P*_HWE_ > 0.05	1478/1791 (11)	1.00 [0.78~1.30]	0.973	0.973
		60.0%	0.010	PCR-RFLP	736/1051 (9)	1.02 [0.77~1.36]	0.898	0.898
AA vs. GG	F	0.0%	0.452	overall	1416/1769 (11)	1.26 [0.93~1.73]	0.151	0.925
		0.0%	0.478	Asian	996/938 (6)	1.65 [0.79~3.46]	0.186	0.973
		13.5%	0.328	Caucasian	420/831 (5)	1.19 [0.84~1.68]	0.339	0.821
		7.2%	0.375	PB	1074/1428 (10)	1.25 [0.91~1.71]	0.172	0.971
		0.0%	0.981	China	776/682 (3)	1.74 [0.44~6.92]	0.433	1.000
		4.9%	0.396	*P*_HWE_ > 0.05	1366/1683 (10)	1.29 [0.93~1.77]	0.125	0.375
		23.6%	0.249	PCR-RFLP	532/819 (7)	1.43 [0.97~2.10]	0.072	0.144
GA vs. GG	F	41.6%	0.071	overall	1416/1769 (11)	1.08 [0.85~1.37]	0.524	0.925
		47.1%	0.092	Asian	996/938 (6)	1.01 [0.70~1.46]	0.945	0.973
		46.8%	0.111	Caucasian	420/831 (5)	1.13 [0.83~1.55]	0.437	0.821
		40.7%	0.086	PB	1074/1428 (10)	1.00 [0.78~1.30]	0.971	0.971
		66.7%	0.049	China	776/682 (3)	1.00 [0.68~1.48]	1.000	1.000
		47.1%	0.049	*P*_HWE_ > 0.05	1366/1683 (10)	1.09 [0.86~1.38]	0.496	0.744
		0.0%	0.437	PCR-RFLP	532/819 (7)	1.45 [0.99~2.14]	0.050	0.144
GA + AA vs. GG	F	44.1%	0.057	overall	1416/1769 (11)	1.12 [0.89~1.40]	0.335	0.925
		54.3%	0.053	Asian	996/938 (6)	1.12 [0.79~1.58]	0.535	0.973
		42.5%	0.138	Caucasian	420/831 (5)	1.12 [0.83~1.50]	0.462	0.821
		43.7%	0.067	PB	1074/1428 (10)	1.05 [0.82~1.34]	0.710	0.971
		65.6%	0.055	China	776/682 (3)	1.05 [0.72~1.53]	0.813	1.000
		49.0%	0.039	*P*_HWE_ > 0.05	1366/1683 (10)	1.13 [0.90~1.41]	0.307	0.614
		16.6%	0.303	PCR-RFLP	532/819 (7)	1.46 [1.03~2.08]	0.035	0.144
AA vs. GG + GA	F	40.7%	0.070	overall	1528/1877 (12)	0.98 [0.79~1.22]	0.866	0.925
		59.1%	0.023	Asian	1108/1046 (7)	0.94 [0.65~1.37]	0.765	0.973
		0.0%	0.430	Caucasian	420/831 (5)	1.00 [0.77~1.30]	0.998	0.998
		45.1%	0.051	PB	1186/1536 (11)	0.98 [0.79~1.21]	0.823	0.971
		24.7%	0.263	China	888/790 (4)	0.50 [0.28~0.88]	0.901	1.000
		46.0%	0.047	*P*_HWE_ > 0.05	1478/1791 (11)	0.99 [0.79~1.23]	0.018	0.108
		60.6%	0.013	PCR-RFLP	644/927 (8)	0.95 [0.75~1.20]	0.680	0.898
carrier A vs. G	F	34.8%	0.112	overall	1528/1877 (12)	1.01 [0.86~1.18]	0.925	0.925
		60.1%	0.020	Asian	1108/1046 (7)	1.00 [0.76~1.30]	0.973	0.973
		0.0%	0.767	Caucasian	420/831 (5)	1.01 [0.83~1.24]	0.887	0.998
		31.5%	0.147	PB	1186/1536 (11)	0.97 [0.82~1.15]	0.737	0.971
		68.3%	0.024	China	888/790 (4)	0.84 [0.61~1.16]	0.285	1.000
		40.6%	0.078	*P*_HWE_ > 0.05	1478/1791 (11)	1.01 [0.86~1.19]	0.895	0.973
		39.5%	0.115	PCR-RFLP	644/927 (8)	1.02 [0.84~1.25]	0.815	0.898

*M* statistical model, *R* random effect, *F* fixed effect, *P*_HWE_ *P*-value of Hardy-Weinberg equilibrium, *P*_heterogeneity_ *P*-value of Cochrane’s Q statistic for the assessment of heterogeneity, *N* Number of included case-control studies, *OR* odds ratio, *CI* confidence interval, *P*_*association*_ *P*-value of association

*BH* Benjamini & Hochberg-adjusted *P*_*association*_

### *VDR rs2228570* polymorphism

**Fig. 4:**
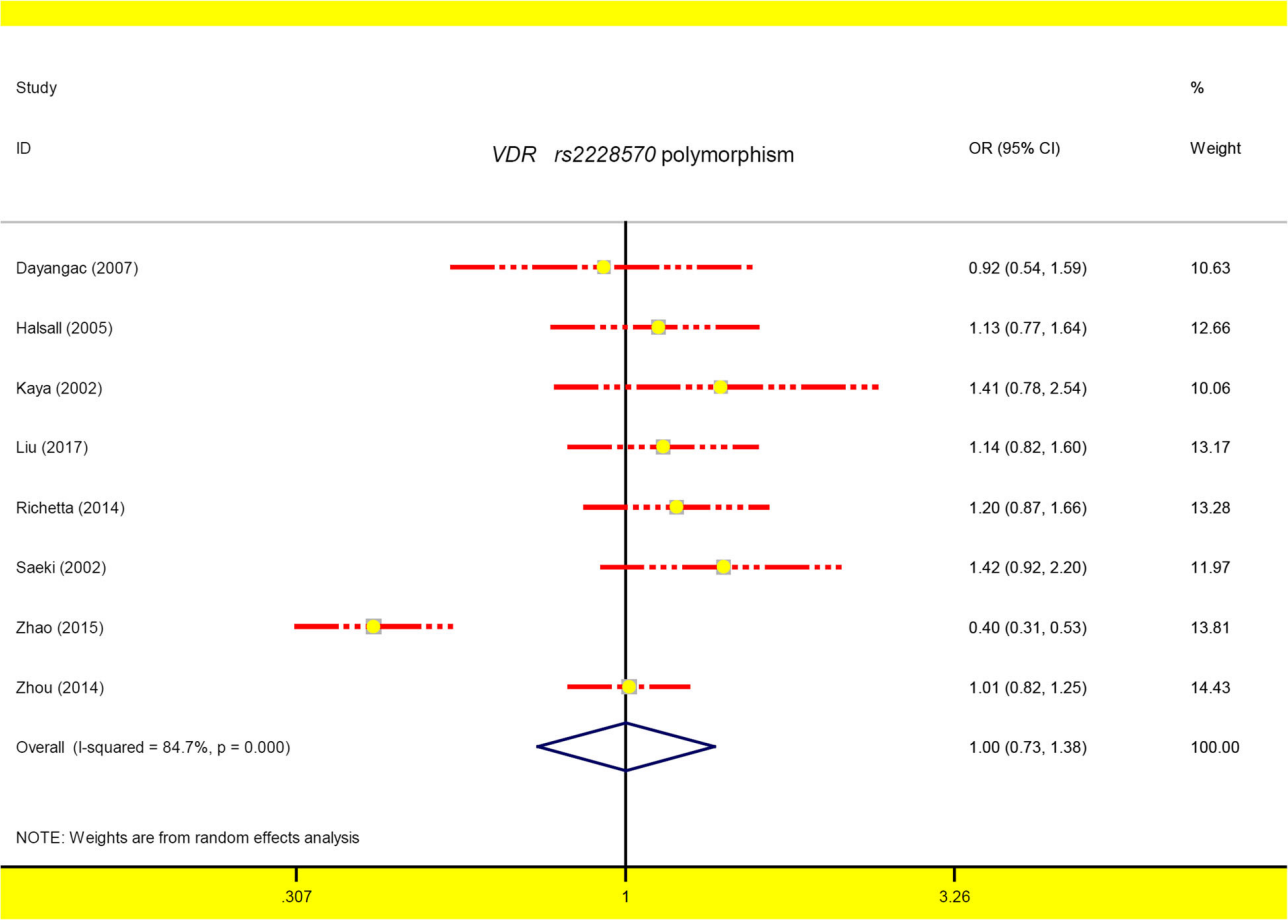
The forest plot for *VDR rs2228570* polymorphism under the allele model

A total of eight studies involving 1308 cases/1253 controls were enrolled for meta-analysis of *VDR rs2228570*. A fixed-effect pooling model was utilized for the TC vs. TT (Table 4, I^2^ = 46.2%, *P*_heterogeneity_ = 0.84), whereas a random-effects pooling model was used for the others (all I^2^ > 50.0%, *P*_heterogeneity_ < 0.05). As shown in Table 4, no statistically significant association was detected in the overall meta-analysis and subsequent subgroup analysis (*P*_*association*_ > 0.05, BH-adjusted *P*_*association*_ > 0.05). Figure 4 shows the forest plot under the allele model, and Additional file 8: Figure S5 and Additional file 9: Figure S6 show the forest plots in the subgroup analysis by the factors of ethnicity and source of controls (allele model). These findings indicated that *VDR rs2228570* might not be associated with the risk of psoriasis.

**Table 4:** Pooled analyses of the association between *VDR rs2228570* polymorphism and susceptibility to psoriasis

Models	M	I^2^	*P*_heterogeneity_	Stratification	case/control (N)	OR [95% CI]	*P*_*association*_	BH
allele C vs. T	R	84.7%	< 0.001	overall	1308/1253 (8)	1.00 [0.73~1.38]	0.989	0.989
		92.2%	< 0.001	Asian	891/751 (4)	0.89 [0.52~1.53]	0.681	0.760
		0.0%	0.766	Caucasian	417/502 (4)	1.16 [0.93~1.43]	0.681	0.681
		88.4%	< 0.001	PB	761/832 (6)	0.99 [0.62~1.58]	0.964	0.987
		93.8	< 0.001	China	776/682 (3)	0.78 [0.41~1.46]	0.429	0.521
		86.6%	< 0.001	*P*_HWE_ > 0.05	1103/1173 (7)	0.99 [0.69~1.42]	0.946	0.955
		0.0%	0.603	PCR-RFLP	424/303 (4)	1.20 [0.95~1.52]	0.121	0.348
CC vs. TT	R	84.4%	< 0.001	overall	1103/1173 (7)	0.96 [0.47~1.97]	0.914	0.989
		90.9%	< 0.001	Asian	891/751 (4)	0.81 [0.29~2.28]	0.695	0.760
		0.0%	0.440	Caucasian	212/422 (3)	1.33 [0.76~2.32]	0.317	0.560
		86.5%	< 0.001	PB	761/832 (6)	0.97 [0.38~2.47]	0.947	0.987
		92.8%	< 0.001	China	776/682 (3)	0.62 [0.19~2.06]	0.438	0.521
		84.4%	< 0.001	*P*_HWE_ > 0.05	1103/1173 (7)	0.96 [0.47~1.97]	0.914	0.955
		6.2%	0.344	PCR-RFLP	219/223 (3)	1.58 [0.78~3.21]	0.204	0.348
TC vs. TT	F	46.2%	0.084	overall	1103/1173 (7)	1.02 [0.84~1.25]	0.810	0.989
		70.4%	0.017	Asian	891/751 (4)	0.96 [0.75~1.21]	0.717	0.760
		0.0%	0.955	Caucasian	212/422 (3)	1.20 [0.84~1.72]	0.325	0.560
		54.3%	0.053	PB	761/832 (6)	0.99 [0.78~1.25]	0.919	0.987
		77.2%	0.012	China	776/682 (3)	0.90 [0.70~1.17]	0.440	0.521
		46.2%	0.084	*P*_HWE_ > 0.05	1103/1173 (7)	1.02 [0.84~1.25]	0.810	0.955
		0.0%	0.886	PCR-RFLP	219/223 (3)	1.25 [0.83~1.89]	0.290	0.348
TC + CC vs. TT	R	76.0%	< 0.001	overall	1103/1173 (7)	1.01 [0.67~1.52]	0.955	0.989
		86.6%	< 0.001	Asian	891/751 (4)	0.90 [0.47~1.74]	0.760	0.760
		0.0%	0.790	Caucasian	212/422 (3)	1.22 [0.87~1.71]	0.253	0.560
		79.7%	< 0.001	PB	761/832 (6)	1.00 [0.60~1.69]	0.987	0.987
		89.5%	< 0.001	China	776/682 (3)	0.77 [0.35~1.71]	0.521	0.521
		76.0%	< 0.001	*P*_HWE_ > 0.05	1103/1173 (7)	1.01 [0.67~1.52]	0.955	0.955
		0.0%	0.651	PCR-RFLP	219/223 (3)	1.30 [0.88~1.92]	0.191	0.348
CC vs. TT + TC	R	79.6%	< 0.001	overall	1103/1173 (7)	0.93 [0.54~1.60]	0.782	0.989
		87.7%	< 0.001	Asian	891/751 (4)	0.82 [0.39~1.71]	0.600	0.760
		0.0%	0.466	Caucasian	212/422 (3)	1.21 [0.72~2.04]	0.467	0.560
		82.0%	< 0.001	PB	761/832 (6)	0.94 [0.46~1.92]	0.869	0.987
		90.0%	< 0.001	China	776/682 (3)	0.68 [0.30~1.55]	0.358	0.521
		79.6%	< 0.001	*P*_HWE_ > 0.05	1103/1173 (7)	0.93 [0.54~1.60]	0.782	0.955
		0.0%	0.396	PCR-RFLP	219/223 (3)	1.41 [0.75~2.68]	0.287	0.348
carrier C vs. T	R	61.8%	0.015	overall	1103/1173 (7)	0.97 [0.76~1.25]	0.840	0.989
		77.9%	0.004	Asian	891/751 (4)	0.91 [0.63~1.32]	0.632	0.760
		0.0%	0.843	Caucasian	212/422 (3)	1.12 [0.84~1.49]	0.444	0.560
		67.2%	0.009	PB	761/832 (6)	0.98 [0.71~1.35]	0.883	0.987
		82.5%	0.003	China	776/682 (3)	0.84 [0.55~1.29]	0.425	0.521
		61.8%	0.015	*P*_HWE_ > 0.05	1103/1173 (7)	0.97 [0.76~1.25]	0.840	0.955
		0.0%	0.772	PCR-RFLP	219/223 (3)	1.17 [0.84~1.63]	0.360	0.360

*M* statistical model, *R* random effect, *F* fixed effect, *P*_HWE_ *P*-value of Hardy-Weinberg equilibrium, *P*_heterogeneity_ *P*-value of Cochrane’s Q statistic for the assessment of heterogeneity, *N* Number of included case-control studies, *OR* odds ratio, *CI* confidence interval, *P*_*association*_ *P*-value of association

*BH* Benjamini & Hochberg-adjusted *P*_*association*_

### *VDR rs731236* polymorphism

**Fig. 5:**
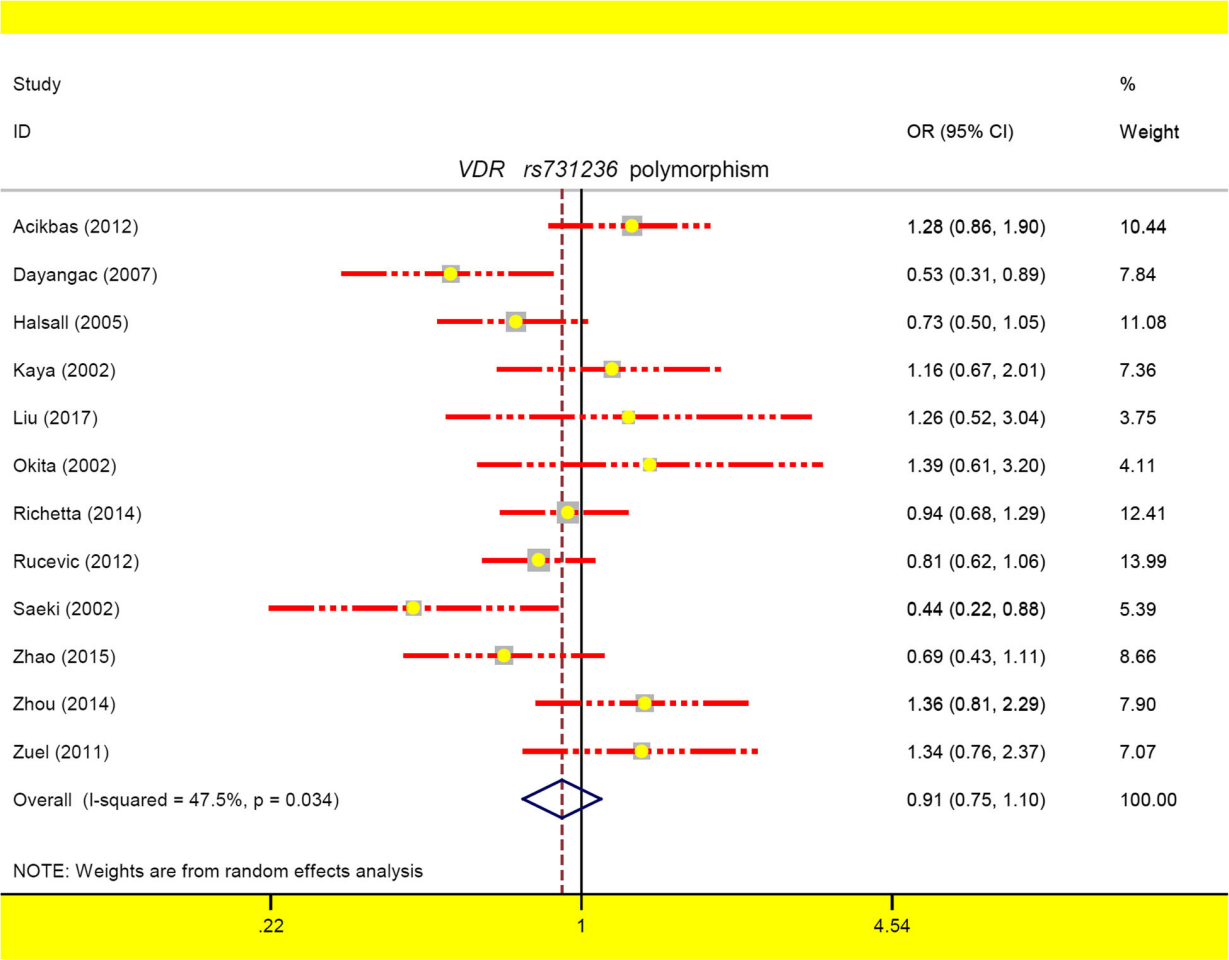
The forest plot for *VDR rs731236* polymorphism under the allele model

During the meta-analysis of *VDR rs731236* containing 1690 cases/1857 controls, a random-effect model was used for the allele C vs. T (*P*_heterogeneity_ = 0.034), TC vs. TT (*P*_heterogeneity_ = 0.043) and TC + CC vs. TT (I^2^ = 50.7%, *P*_heterogeneity_ = 0.027), and a fix-effect model was applied for others (all I^2^ < 50.0%, *P*_heterogeneity_ > 0.05). As shown in Table 5, no differences between cases and controls were detected in all analyses (Table 5, all *P*_*association*_ > 0.05, BH-adjusted *P*_*association*_ *>* 0.05). Figure 5 presents the forest plot of the allele model, and Additional file 10: Figure S7 and Additional file 11: Figure S8 show the forest plot in the subgroup analysis by the factors of ethnicity and source of controls (allele model). As a result, *VDR rs731236* polymorphism is not significantly associated with the odds of psoriasis disease.

**Table 5:** Pooled analyses of the association between *VDR rs731236* polymorphism and susceptibility to psoriasis

Models	M	I^2^	*P*_heterogeneity_	Stratification	case/control (N)	OR [95% CI]	*P*_*association*_	BH
allele C vs. T	R	47.5%	0.034	overall	1690/1857 (12)	0.91 [0.75~1.10]	0.325	0.690
		57.2%	0.053	Asian	941/837 (5)	0.91 [0.58~1.43]	0.689	0.798
		47.4%	0.090	Caucasian	699/970 (6)	0.87 [0.70~1.08]	0.216	0.629
		47.7%	0.045	PB	1143/1436 (10)	0.90 [0.73~1.12]	0.341	0.524
		49.7%	0.137	China	776/682 (3)	1.01 [0.62~1.64]	0.962	0.974
		73.1%	0.024	Turkey	206/256 (3)	0.93 [0.54~1.61]	0.806	0.824
		45.7%	0.056	*P*_HWE_ > 0.05	1383/1675 (10)	0.90[0.72~1.11]	0.324	0.389
		57.4%	0.021	PCR-RFLP	806/907 (8)	0.88 [0.68~1.14]	0.360	0.744
CC vs. TT	F	38.2%	0.114	overall	1325/1508 (9)	0.92[0.67~1.25]	0.581	0.690
		0.0%	0.460	Asian	781/568 (3)	0.80[0.24~2.66]	0.717	0.798
		58.7%	0.046	Caucasian	494/890 (5)	0.87 [0.62~1.22]	0.419	0.629
		43.7%	0.087	PB	983/1167 (8)	0.90 [0.66~1.24]	0.524	0.524
		73.8%	0.022	Turkey	206/256 (3)	1.05 [0.68~1.81]	0.868	0.974
		0.0%	0.511	China	666/499 (2)	1.24 [0.28~5.53]	0.775	0.824
		24.8%	0.231	*P*_HWE_ > 0.05	1223/1406 (8)	0.79[0.56~1.12]	0.183	0.386
		59.8%	0.029	PCR-RFLP	551/741 (6)	0.88 [0.61~1.28]	0.499	0.749
TC vs. TT	R	46.8%	0.043	overall	1485/1777 (11)	0.95[0.72~1.24]	0.690	0.690
		51.5%	0.083	Asian	941/837 (5)	0.90[0.58~1.42]	0.658	0.798
		61.0%	0.036	Caucasian	494/890 (5)	0.98[0.63~1.51]	0.918	0.918
		46.6%	0.051	PB	1143/1436 (10)	0.91[0.68~1.22]	0.523	0.524
		50.4%	0.133	China	776/682 (3)	0.97[0.58~1.63]	0.915	0.974
		73.1%	0.024	Turkey	206/256 (3)	1.26 [0.53~2.99]	0.593	0.824
		15.7%	0.299	*P*_HWE_ > 0.05	1383/1675 (10)	0.85[0.69~1.06]	0.155	0.386
		56.3%	0.033	PCR-RFLP	601/827 (7)	0.88 [0.61~1.28]	0.988	0.988
TC + CC vs. TT	R	50.7%	0.027	overall	1485/1777 (11)	0.94[0.71~1.23]	0.636	0.690
		56.0%	0.059	Asian	941/837 (5)	0.90[0.57~1.44]	0.671	0.798
		61.4%	0.035	Caucasian	494/890 (5)	0.93[0.62~1.40]	0.733	0.880
		49.6%	0.037	PB	1143/1436 (10)	0.90 [0.67~1.19]	0.453	0.524
		51.4%	0.128	China	776/682 (3)	0.99[0.59~1.66]	0.974	0.974
		76.8%	0.013	Turkey	206/256 (3)	1.12 [0.47~2.68]	0.794	0.824
		32.9%	0.145	*P*_HWE_ > 0.05	1383/1675 (10)	0.86[0.67~1.09]	0.205	0.386
		61.7%	0.016	PCR-RFLP	601/827 (7)	0.96 [0.63~1.45]	0.843	0.988
CC vs. TT + TC	F	4.4%	0.398	overall	1325/1508 (9)	0.91[0.69~1.20]	0.487	0.690
		0.0%	0.506	Asian	781/568 (3)	0.85[0.26~2.85]	0.798	0.798
		25.0%	0.254	Caucasian	494/890 (5)	0.86[0.64~1.16]	0.330	0.629
		10.9%	0.345	PB	983/1167 (8)	0.90[0.68~1.19]	0.442	0.524
		0.0%	0.543	China	666/499 (2)	1.30[0.29~5.76]	0.734	0.974
		47.7%	0.148	Turkey	206/256 (3)	0.81 [0.52~1.27]	0.361	0.824
		16.3%	0.301	*P*_HWE_ > 0.05	1223/1406 (8)	0.89[0.64~1.23]	0.472	0.472
		27.6%	0.228	PCR-RFLP	551/741 (6)	0.82 [0.59~1.14]	0.228	0.744
carrier C vs. T	F	1.1%	0.430	overall	1485/1777 (11)	0.93[0.80~1.09]	0.380	0.690
		41.4%	0.145	Asian	941/837 (5)	0.92[0.68~1.23]	0.558	0.798
		0.0%	0.617	Caucasian	494/890 (5)	0.92[0.76~1.11]	0.388	0.629
		0.0%	0.492	PB	1143/1436 (10)	0.90[0.77~1.06]	0.223	0.524
		37.9%	0.200	China	776/682 (3)	0.98[0.70~1.38]	0.922	0.974
		16.6%	0.302	Turkey	206/256 (3)	0.96[0.70~1.32]	0.824	0.824
		2.6%	0.415	*P*_HWE_ > 0.05	1383/1675 (10)	0.91[0.77~1.07]	0.257	0.386
		11.6%	0.341	PCR-RFLP	601/827 (7)	0.92 [0.75~1.11]	0.372	0.744

*M* statistical model, R random effect, F fixed effect, *P*_HWE_ *P*-value of Hardy-Weinberg equilibrium, *P*_heterogeneity_ *P*-value of Cochrane’s Q statistic for the assessment of heterogeneity, *N* Number of included case-control studies, *OR* odds ratio, *CI* confidence interval, *P*_*association*_ *P*-value of association,

BH Benjamini & Hochberg-adjusted P_association_

### Sensitivity analysis and publication bias

**Fig. 6:**
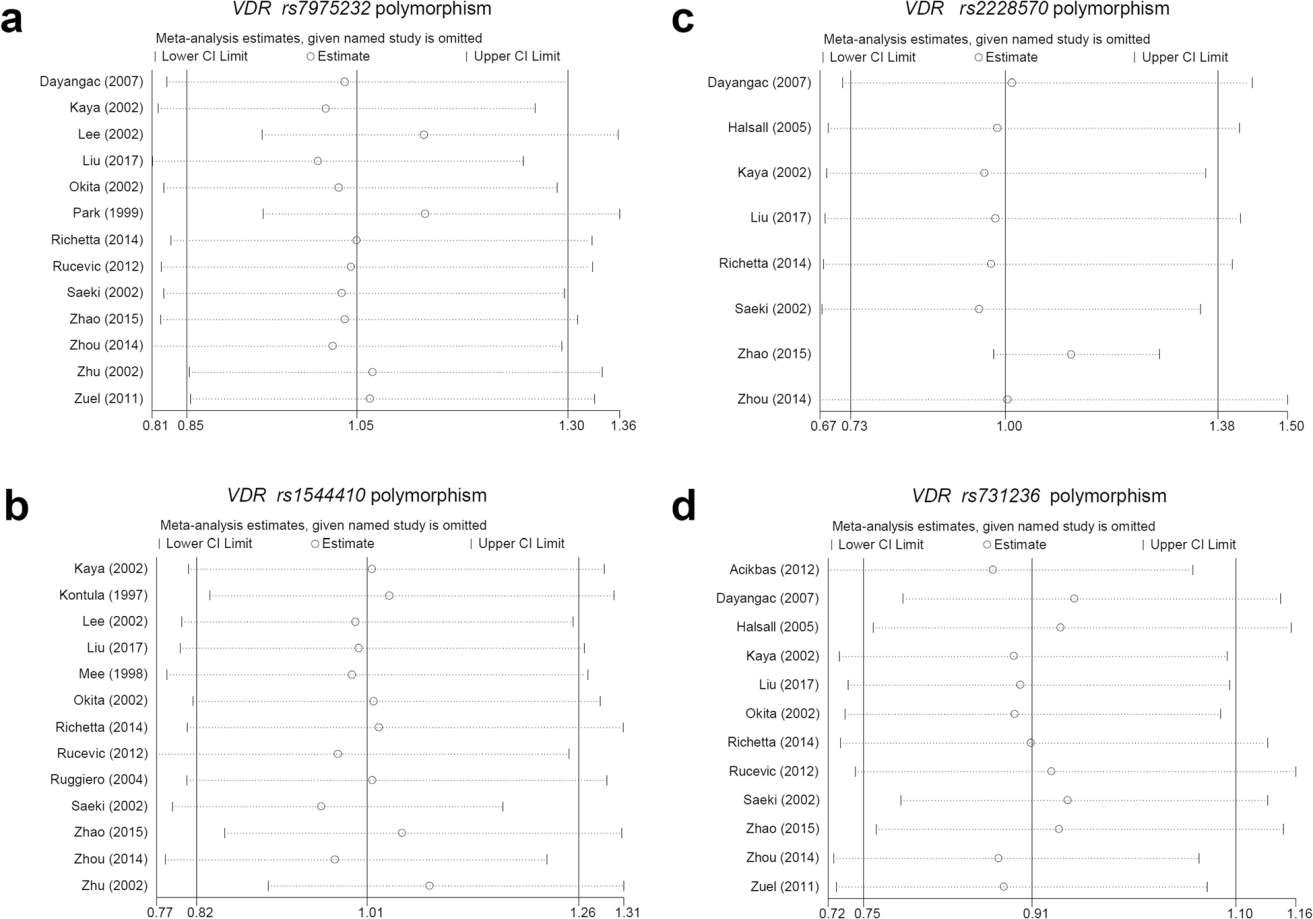
Sensitivity analysis result under the allele model. **a** *rs7975232* polymorphism; **b** *rs1544410* polymorphism; **c** *rs2228570* polymorphism; **d** *rs731236* polymorphism

We did not observe largely altered meta-analysis estimates in the results of our sensitivity analysis (Fig. 6 for the allele model; and other data not shown), suggesting the statistical reliability of pooling results. We also conducted the Begg’s and Egger’s tests to assess the potential publication bias. As shown in Table 6, the *P-*value of Begg’s and Egger’s test was greater than 0.05 under all the above genetic models. Additional file 12: Figure S9 and Additional file 13: Figure S10 show the Begg’s funnel plots and Egger’s publication bias plots under the allele model. We observed basically symmetrical funnel plots. Therefore, there is no large publication bias in our study.

**Table 6:** Publication bias assessments

polymorphism	Models	Begg’s test		Egger’s test	
		z	*P*_*Begg*_	t	*P*_*Egger*_
*rs7975232*	allele C vs. A	0.43	0.669	−1.10	0.296
	CC vs. AA	1.16	0.246	−1.02	0.331
	AC vs. AA	1.04	0.300	−1.24	0.241
	AC + CC vs. AA	1.40	0.161	−1.48	0.167
	CC vs. AA+AC	0.18	0.855	0.35	0.736
	carrier C vs. A	0.67	0.502	−1.11	0.291
*rs1544410*	allele A vs. G	0.31	0.760	−0.72	0.487
	AA vs. GG	0.00	1.000	−0.44	0.669
	GA vs. GG	0.47	0.640	−0.22	0.832
	GA + AA vs GG	0.00	1.000	−0.13	0.896
	AA vs. GG + GA	0.07	0.945	0.04	0.966
	carrier A vs. G	−0.07	1.000	−0.35	0.735
*rs2228570*	allele C vs. T	0.62	0.536	0.83	0.437
	CC vs. TT	0.30	0.764	0.66	0.539
	TC vs. TT	0.00	1.000	0.24	0.823
	TC + CC vs. TT	0.00	1.000	0.30	0.777
	CC vs. TT + TC	0.90	0.368	0.95	0.387
	carrier C vs. T	0.60	0.548	0.70	0.515
*rs731236*	allele C vs. T	0.07	0.945	0.53	0.611
	CC vs. TT	−0.10	1.000	−0.14	0.895
	TC vs. TT	0.62	0.533	1.13	0.286
	TC + CC vs. TT	0.62	0.533	1.08	0.310
	CC vs. TT + TC	0.10	0.917	−0.27	0.795
	carrier C vs. T	0.16	0.876	0.43	0.675

*P*_*Begg*_ *P*-value of Begg’s test, *P*_*Egger*_ *P*-value of Egger’s test

## Discussion

In the current study, we searched eight online electronic databases, including PubMed, EMBASE, WOS, CNKI, WANFANG, OVID, Scopus and Cochrane (up to August 18, 2019), to enroll a total of 18 case-control studies. Based on the currently available data, we conducted a series of overall meta-analysis and subgroup analysis to evaluate the genetic relationship regarding *VDR rs7975232*, *rs1544410*, *rs2228570*, and *rs731236* polymorphisms and psoriasis susceptibility. Here, we used the “RS” naming, the most common polymorphism nomenclature in the single nucleotide polymorphism database (dbSNP), rather than the name of restriction enzymes in polymerase chain reaction-restriction fragment length polymorphism (PCR-RFLP) assay, namely A*pa*I, B*sm*I, F*ok*I, and TaqI. Moreover, six genetic models, including allele, homozygote, heterozygote, dominant, recessive, and carrier models, were employed. BH correction method was also utilized to adjust the *P*-values obtained from the multiple comparisons.

In our updated meta-analysis of *VDR rs7975232*, we enrolled thirteen case-control studies for pooling and did not detect any significant statistical association between the *VDR rs7975232* polymorphism and the odds of psoriasis. In 2012, Lee, YH et al. included six case-control studies [14, 16–18, 21, 24] for a meta-analysis regarding the association between the *VDR rs7975232* polymorphism and psoriasis susceptibility [31]. Data from the “Turkish” subgroup containing two case-control studies [16, 17] indicated a potential genetic correlation between the *VDR rs7975232* polymorphism and psoriasis susceptibility [31]. In 2013, Liu, J. L. et al. included eight case-control studies [14, 16–18, 20, 21, 24, 25] for an updated meta-analysis and only found a positive result under the dominant model (*P*_*association*_ = 0.043) but not other genetic models [5]. In 2013, Stefanic, M. et al. performed another meta-analysis, which did not include one study [14] but added another study [13], and reported no robust correlation between the *VDR rs7975232* polymorphism and psoriasis risk [4]. In the present meta-analysis, we added four new studies [15, 19, 29, 30] in the overall population and subgroup meta-analyses based on the factors of the control source, ethnicity, country, HWE and genotyping method under six genetic models. Our data failed to support the essential role of the *VDR rs7975232* polymorphism in the odds of psoriasis, which is in line with the data of Lee, YH [3]..

For *rs1544410*, *rs2228570*, and *rs731236* polymorphisms, compared with three previous meta-analyses [4, 5, 31], we added four new eligible studies [15, 19, 29, 30] in our updated meta-analysis. Nevertheless, no statistically significant conclusions between *VDR rs1544410, rs2228570* and *VDR rs731236* polymorphisms and psoriasis susceptibility were observed. The conclusions regarding the genetic effect of *VDR rs1544410, rs2228570,* but not *VDR* rs731236 polymorphisms on the odds of psoriasis disease were consistent with the pooling results of Lee, YH [3]., which contains sixteen studies [13, 14, 16–22, 24–30]. Subgroup analysis of “Caucasian” suggested that the *VDR rs731236* polymorphism is linked to the risk of psoriasis in the Caucasian population under the recessive model, but not the allele, homozygote and dominant models [3]. In our updated study, we added another two new studies [15, 23], and applied two more models, including heterozygote and carrier models. Apart from ethnicity, we also considered the factors of control source, country, and HWE in the subgroup analyses. However, no positive conclusion was observed in any comparison of *VDR rs731236*. The potential slight genetic effect of *VDR rs731236* polymorphism in the high susceptibility to psoriasis in the Caucasian population was masked by the adding of more sample size, and the utilization of BH correction of *P*-value. Despite of this, we cannot exclude the *VDR rs731236* polymorphism in the odds of psoriasis in the Caucasian population, the support of more case-control studies is required.

In this study, three investigators tried the best to reduce the potential bias during database retrieval, study selection, data extraction, and statistical analysis. However, some limitations should be addressed. First, less than ten case-control studies were included in the meta-analysis of the *VDR rs2228570* in the overall population. In addition, only one case-control study of the African population [21] is included in the subgroup analysis of *VDR rs7975232* and *rs731236* by the factor of ethnicity. Given the lack of sufficient genotype data, we did not detect the potential genetic influence of the other *VDR* variants (such as rs4516035) or the combined variants of *VDR* and other relevant genes. Second, high heterogeneity between studies was detected in some analyses of *VDR* polymorphisms and psoriasis susceptibility. We observed a decreased level of between-study heterogeneity in some subgroups of “Asian” or “Caucasian”, indicating that the factor of ethnicity may be implicated in the source of heterogeneity. Third, conflicting conclusions regarding the potential role of *VDR* polymorphisms in the partial resistance of psoriasis patients to calcipotriol therapy were reported [15, 16, 23, 26, 27]. We extracted the basic information regarding the gender, age, calcipotriol response, and family history within the included case-control studies; nevertheless, the lack of sufficient data did not support the preformation of the relevant stratification analysis or adjusted effect estimates. Increased sample sizes are still needed to investigate the genetic relationship between different *VDR* polymorphisms and the response of psoriasis patients to drug treatments.

## Conclusions

Above all, based on the presently available case-control studies, our pooling analysis data and previous reports do not provide the robust statistical evidence linking *VDR rs7975232*, *rs1544410*, and *rs2228570* polymorphisms with the odds of psoriasis. More case-control studies will be of assistance to us to further confirm the effect of the *VDR* polymorphisms on the psoriasis susceptibility in the Caucasian population.

## Supplementary information

**Additional file 1: Table S1.** Searching terms for our meta-analysis (up to August 18, 2019).

**Additional file 2: Table S2.** The clinical characteristics of included case-control studies.

**Additional file 3: Table S3.** Quality assessment of included case-control studies.

**Additional file 4: Figure S1.** The forest plot for *VDR rs797523*2 polymorphism in the subgroup analysis by ethnicity under the allele model.

**Additional file 5: Figure S2.** The forest plot for *VDR rs797523*2 polymorphism in the subgroup analysis by the source of controls under the allele model.

**Additional file 6: Figure S3.** The forest plot for *VDR rs1544410* polymorphism in the subgroup analysis by ethnicity under the allele model.

**Additional file 7: Figure S4.** The forest plot for *VDR rs1544410* polymorphism in the subgroup analysis by the source of controls under the allele model.

**Additional file 8: Figure S5.** The forest plot for *VDR rs2228570* polymorphism in the subgroup analysis by ethnicity under the allele model.

**Additional file 9: Figure S6.** The forest plot for *VDR rs2228570* polymorphism in the subgroup analysis by the source of controls under the allele model.

**Additional file 10: Figure S7.** The forest plot for *VDR rs731236* polymorphism in the subgroup analysis by ethnicity under the allele model.

**Additional file 11: Figure S8.** The forest plot for *VDR rs731236* polymorphism in the subgroup analysis by the source of controls under the allele model.

**Additional file 12: Figure S9.** Publication bias of *VDR rs7975232* and *rs1544410* polymorphism under the allele model. a-b *rs7975232* polymorphism; c-d *rs1544410* polymorphism.

**Additional file 13: Figure S10.** Publication bias of *VDR rs2228570* and *rs731236* polymorphism under the allele model. a-b *rs2228570* polymorphism; c-d *rs*731236 polymorphism.

## Data Availability

All data generated or analyzed during the present study are included in this published article.

## Abbreviations

BH: Benjamini & Hochberg
CNKI: China National Knowledge Infrastructure
dbSNP: single nucleotide polymorphism database
EMBASE: Excerpta Medica Database
HWE: Hardy-Weinberg equilibrium
LDR: Ligase detection reactions
NOS: Newcastle-Ottawa quality assessment scale
ORs: Odd ratios
PCR-RFLP: Polymerase chain reaction-restriction fragment length polymorphism
SNP: Single nucleotide polymorphism
VDR: Vitamin D Receptor
WOS: Web of Science
