# Supplementary material for: Pooling analysis regarding the impact of human vitamin D receptor variants on the odds of psoriasis
Source: BMC Med Genet. 2019 Oct 17;20:161. doi: 10.1186/s12881-019-0896-6 (PMC6796361; doi:10.1186/s12881-019-0896-6)
Supplement: Supplementary file 1 — Additional file 1: Table S1. Searching terms for our meta-analysis (up to August 18, 2019). [file 12881_2019_896_MOESM1_ESM.docx]

Table S1 Searching terms for our meta-analysis (up to August 18, 2019).

| **Database** | **Step** | **Search terms** | **Number of articles** |
| --- | --- | --- | --- |
| **Pubmed** | #1 | "Receptors, Calcitriol"[Mesh] | **7,339** |
|  | #2 | ((((((((((((((((((((((((((((((((((((1,25-Dihydroxycholecalciferol Receptors) OR 1,25 Dihydroxycholecalciferol Receptors) OR Receptors, 1,25-Dihydroxycholecalciferol) OR Vitamin D3 Receptor) OR D3 Receptor, Vitamin) OR Receptor, Vitamin D3) OR Vitamin D3 Receptors) OR Receptors, Vitamin D3) OR D3 Receptors, Vitamin) OR 1,25-Dihydroxyvitamin D3 Receptor) OR 1,25 Dihydroxyvitamin D3 Receptor) OR D3 Receptor, 1,25-Dihydroxyvitamin) OR Receptor, 1,25-Dihydroxyvitamin D3) OR 1,25-Dihydroxyvitamin D3 Receptors) OR 1,25 Dihydroxyvitamin D3 Receptors) OR D3 Receptors, 1,25-Dihydroxyvitamin) OR Receptors, 1,25-Dihydroxyvitamin D3) OR Calcitriol Receptor) OR Receptor, Calcitriol) OR Calcitriol Receptors) OR Receptors, Cholecalciferol) OR Cholecalciferol Receptors) OR Vitamin D Receptor) OR D Receptor, Vitamin) OR Receptor, Vitamin D) OR Receptors, Vitamin D) OR D Receptors, Vitamin) OR Vitamin D Receptors) OR 1,25-Dihydroxyvitamin D 3 Receptor) OR 1,25 Dihydroxyvitamin D 3 Receptor) OR Receptors, 1,25-Dihydroxyvitamin D 3) OR Receptors, Vitamin D 3) OR Vitamin D 3 Receptor) OR Vitamin D 3 Receptors) OR 1,25-Dihydroxycholecalciferol Receptor) OR 1,25 Dihydroxycholecalciferol Receptor) OR Receptor, 1,25-Dihydroxycholecalciferol | **12,857** |
|  | #3 | #1 OR #2 | **12,857** |
|  | #4 | "Psoriasis"[Mesh] | **38,053** |
|  | #5 | ((((Psoriases) OR (Pustulosis of Palms and Soles)) OR Pustulosis Palmaris et Plantaris) OR Palmoplantaris Pustulosis) OR (Pustular Psoriasis of Palms and Soles) | **48,986** |
|  | #6 | #4 OR #5 | **48,986** |
|  | #7 | #3 AND #6 | **251** |
| **WOS** | #1 | TOPIC: (Psoriasis) OR TOPIC: (Psoriases) OR TOPIC: (Pustulosis of Palms and Soles) OR TOPIC: (Pustulosis Palmaris et Plantaris) OR TOPIC: (Palmoplantaris Pustulosis) OR TOPIC: (Pustular Psoriasis of Palms and Soles) | **58,872** |
|  | #2 | TOPIC: (Receptors, Calcitriol) OR TOPIC: (1,25-Dihydroxycholecalciferol Receptors) OR TOPIC: (1,25 Dihydroxycholecalciferol Receptors) ORTOPIC: (Receptors, 1,25-Dihydroxycholecalciferol) OR TOPIC: (Vitamin D3 Receptor) OR TOPIC: (D3 Receptor, Vitamin) OR TOPIC: (Vitamin D3 Receptors) OR TOPIC: (Calcitriol Receptor) OR TOPIC: (Receptors, 1,25-Dihydroxyvitamin D3) OR TOPIC: (D3 Receptor, 1,25-Dihydroxyvitamin) ORTOPIC: (Vitamin D Receptors) OR TOPIC: (D Receptors, Vitamin) OR TOPIC: (VDR)  Databases= WOS, KJD, RSCI, SCIELO Timespan=All years  Search language=Auto | **28,265** |
|  | #3 | #1 AND #2 | **451** |
| **Embase** | #1 | 'vitamin d receptor'/exp OR 'vitamin d receptor' OR 'vdr' OR 'vitamin d receptors' OR 'vitamin d (1,25- dihydroxyvitamin d3) receptor' OR '1,25-dihydroxyvitamin d3 receptor'/exp OR '1,25-dihydroxyvitamin d3 receptor' | **15,925** |
|  | #2 | 'psoriasis'/exp OR 'psoriasis' OR 'psoriasiform dermatitis'/exp OR 'psoriasiform dermatitis' OR 'psoriasiform dermatosis'/exp OR 'psoriasiform dermatosis' OR 'psoriasiform lesion'/exp OR 'psoriasiform lesion' OR 'psoriasiform rash'/exp OR 'psoriasiform rash' OR 'psoriatic epidermis'/exp OR 'psoriatic epidermis' OR 'psoriatic skin'/exp OR 'psoriatic skin' OR 'skin rash'/exp OR 'skin rash' OR 'psoriasiform' OR 'willan lepra'/exp OR 'willan lepra' | **199,066** |
|  | #3 | #1 AND #2 | **342** |
| **CNKI** | #1 | SU=' VDR' OR SU=' psoriasis' | **54** |
| **WANFANG** | #1 | VDR* psoriasis | **6** |
| **OVID** | #1 | ('vitamin D receptor' OR 'VDR' OR 'Vitamin D Receptors').af. | **26,504** |
|  | #2 | ('psoriasis' OR 'psoriasiform dermatitis' OR 'psoriasiform dermatosis' OR 'psoriasiform lesion' OR 'psoriasiform rash' OR 'psoriatic epidermis' OR 'psoriatic skin' OR 'skin rash' OR 'psoriasiform' OR 'willan lepra').af. | **149,472** |
|  | #3 | #1 AND #2 | **1,127** |
|  | #4 | limit 3 to yr="1860 -Current" [Limit not valid in DARE; records were retained] | **1,127** |
|  | #5 | limit 4 to humans [Limit not valid in AMED,Books@Ovid,Your Journals@Ovid,Joanna Briggs Institute EBP Database -,ACP Journal Club,CCTR,CDSR,CCA,CLCMR,DARE,Journals@Ovid; records were retained] | **1,110** |
|  | #6 | limit 5 to original articles [Limit not valid in AMED,Books@Ovid,Joanna Briggs Institute EBP Database -,ACP Journal Club,CCTR,CDSR,CCA,CLCMR,DARE,CLHTA,CLEED,Ovid MEDLINE(R),Ovid MEDLINE(R) Daily Update,Ovid MEDLINE(R) In-Process,Ovid MEDLINE(R) Publisher; records were retained] | **684** |
| **Scopus** | #1 | (TITLE-ABS-KEY ("Receptors, Calcitriol") OR TITLE-ABS-KEY ("1,25-Dihydroxycholecalciferol Receptors") OR TITLE-ABS-KEY ("1,25 Dihydroxycholecalciferol Receptors") OR TITLE-ABS-KEY ("Receptors, 1,25-Dihydroxycholecalciferol") OR TITLE-ABS-KEY ("Vitamin D3 Receptor") OR TITLE-ABS-KEY ("D3 Receptor, Vitamin") OR TITLE-ABS-KEY ("Vitamin D3 Receptors") OR TITLE-ABS-KEY ("Calcitriol Receptor") OR TITLE-ABS-KEY ("Receptors, 1,25-Dihydroxyvitamin D3") OR TITLE-ABS-KEY ("D3 Receptor, 1,25-Dihydroxyvitamin") OR TITLE-ABS-KEY ("Vitamin D Receptors") OR TITLE-ABS-KEY ("D Receptors, Vitamin") OR TITLE-ABS-KEY (vdr)) AND DOCTYPE (ar) | **9,672** |
|  | #2 | (TITLE-ABS-KEY (psoriasis) OR TITLE-ABS-KEY (psoriases) OR TITLE-ABS-KEY ("Pustulosis of Palms and Soles") OR TITLE-ABS-KEY ("Pustulosis Palmaris et Plantaris") OR TITLE-ABS-KEY ("Palmoplantaris Pustulosis") OR TITLE-ABS-KEY ("Pustular Psoriasis of Palms and Soles")) | **68,326** |
|  | #3 | #1 AND #2 | **141** |
| **Cochrane** | #1 | (vitamin D receptor) OR (VDR) OR (Vitamin D Receptors) OR (D3 Receptors, Vitamin) OR (vitamin D(3) receptor) | **1,240** |
|  | #2 | (Psoriasis) OR (Psoriases) OR (Palmoplantaris Pustulosis) OR (Pustulosis Palmaris et Plantaris) OR (Pustulosis of Palms and Soles) | **7,216** |
|  | #3 | #1 AND #2 | **26** |

Note: *WOS* Web of Science, *Embase* ExcerptaMedica Database, *CNKI* China National Knowledge Infrastructure
