# Supplementary material for: Pooling analysis regarding the impact of human vitamin D receptor variants on the odds of psoriasis
Source: BMC Med Genet. 2019 Oct 17;20:161. doi: 10.1186/s12881-019-0896-6 (PMC6796361; doi:10.1186/s12881-019-0896-6)
Supplement: Supplementary file 2 — Additional file 2: Table S2. The clinical characteristics of included case-control studies. [file 12881_2019_896_MOESM2_ESM.docx]

Table S2 The clinical characteristics of included case-control studies.

| **First author** | **Year** | **Case** | | | | | | | **Control** | | | |
| --- | --- | --- | --- | --- | --- | --- | --- | --- | --- | --- | --- | --- |
|  |  | **Total** | **Gender (M/F)** | **Age** | **Details** | **Age onset** | **Calcipotriol^&^** | **Family history (positive)** | **Total** | **Gender (M/F)** | **Age** | **Details** |
| **Acikbas** | **2012** | 102 | 55/47 | 44.4±16.4 | 69 with plaque; 16 with plaque plus guttate; 10 with guttate; 5 with palmoplantar; 2 with pustular palmoplantar type of psoriasis | 32.3±16.8 | 31/18 | 14 | 102 | 52/50 | 40.8±16.9 | healthy controls |
| **Dayangac** | **2007** | 51 | 22/29 | 38.5 | 45 with psoriasis vulgaris and 6 with psoriatic arthritis | <40 (n=49) | 22/25 | 51 | 100 | 52/48 | 18 | healthy subjects |
| **Halsall** | **2005** | 205 | 119/86 | 44 | psoriasis received topical calcipotriol treatment | 23 | 61/137 | 88 | 80 | 41/39 | 56 | patients attending the hospital who had no inflammatory skin disease or known malignancy. |
| **Kaya** | **2002** | 53 | 25/28 | 40.6±19.9 | 38 with psoriasis vulgaris; 10 with guttate psoriasis | NA | NA | NA | 54 | 19/35 | 43.2±13.1 | unrelated healthy controls |
| **Kontula** | **1997** | 19 | NA | NA | psoriasis | NA | 9/10 | NA | 75 | NA | NA | healthy blood donors |
| **Lee** | **2002** | 55 | 29/26 | 36.4±16.3 | psoriasis vulgaris receiving topical calcipotriol therapy | 25.5±15.8 | 14/29 | 14 | 104 | NA | 24.5 | unrelated healthy individuals |
| **Liu** | **2017** | 110 | 67/43 | 35.3±15.0 | 75 PASI scores less than 10 were topically treated with caleipotriol ointment alone | NA | 19/53 | 110 | 183 | 104/79 | 33.8±13.3 | healthy controls |
| **Mee** | **1998** | 92 | NA | NA | 92 patients with chronic plaque psoriasis | NA | 19/73 | NA | 124 | NA | NA | ethnically matched, local control population of 124 individuals |
| **Okita** | **2002** | 50 | 35/15 | 51.1 | 38 with psoriasis vulgaris; 6 with psoriasis pustulosa; 1 with psoriasis arthropathica; 4 with pustulosis palmaris et plantaris and 1 with acrodermatitis continua | <40 (n=24) | NA | NA | 86 | 36/50 | 51 | normal controls |
| **Park** | **1999** | 104 | 52/52 | 37.1±15.3 | All patients had psoriasis vulgaris | 24.9±14.6 | NA | NA | 104 | NA | NA | healthy controls |
| **Richetta** | **2014** | 108 | 61/47 | NA | 38 with arthropathic | 37.8 | NA | 34 | 268 | 197/71 | 53.4 | healthy controls |
| **Richetta** | **2012** | 180 | 87/93 | NA | patients with chronic plaque psoriasis | 39.2±17.6 | NA | NA | 366 | 117/249 | NA | ethnically matched, healthy controls of the Croatian origin |
| **Ruggiero** | **2004** | 60 | 46/14 | 47.3 | all with typical sharply-defined, dull-red scaly plaques located on extensor surfaces of limbs | <40 (n=38) | NA | NA | 68 | 50/18 | 57.2 | healthy subjects |
| **Saeki** | **2002** | 115 | 74/41 | 51.8±16.0 | psoriasis vulgaris | NA | 30/24 | NA | 69 | 43/26 | 31.3±9.3 | healthy individuals |
| **Zhao** | **2015** | 324 | 232/92 | 42.0±13.5^a^ 40.9±11.9^b^ | 125 mild and 199 moderate to severe psoriatic patients | 35.9±10.7^a^34.4±13.2^b^ | 89/36 | 29 | 158 | 112/46 | 42.6±10.3 | healthy age- and gender-matched volunteers |
| **Zhou** | **2014** | 342 | 209/133 | 33.6±13.7 | Psoriasis confirmed by clinical phenomenon and histology | NA | NA | NA | 341 | 200/141 | 33.2±14.5 | patients did not have a history of systemic, infectious, autoimmune, genetic, atopic or malignant diseases |
| **Zhu** | **2002** | 112 | 56/56 | 41.3±10.1 | psoriasis vulgaris | 30.7±11.4 | NA | 30 | 108 | 54/54 | 48.7±23.4 | healthy controls |
| **Zuel** | **2011** | 50 | 31/19 | 41.2±15.6 | psoriasis vulgaris | 32.4±14.2 | NA | NA | 50 | 34/16 | 35.2±7.6 | healthy controls |

Note: *M* Males, *F* Females, *NA* not available, *PASI* psoriasis area and severity index, *&* non-responders/responders, *a* for calcipotriol monotherapy, *b* for calcipotriol plus acitretin
