# Supplementary material for: Pooling analysis regarding the impact of human vitamin D receptor variants on the odds of psoriasis
Source: BMC Med Genet. 2019 Oct 17;20:161. doi: 10.1186/s12881-019-0896-6 (PMC6796361; doi:10.1186/s12881-019-0896-6)
Supplement: Supplementary file 3 — Additional file 3: Table S3. Quality assessment of included case-control studies. [file 12881_2019_896_MOESM3_ESM.docx]

Table S3 Quality assessment of included case-control studies

| **Number** | **First author** | **Year** | **NOS score** | **Term 1** | **Term 2** | **Term 3** | **Term 4** | **Term 5** | **Term 6** | **Term 7** | **Term 8** | **Term 9** |
| --- | --- | --- | --- | --- | --- | --- | --- | --- | --- | --- | --- | --- |
| **1** | **Acikbas** | **2012** | **8** | **1** | **1** | **1** | **1** | **1** | **1** | **0** | **1** | **1** |
| **2** | **Dayangac** | **2007** | **7** | **0** | **1** | **1** | **1** | **0** | **1** | **1** | **1** | **1** |
| **3** | **Halsall** | **2005** | **5** | **1** | **1** | **0** | **0** | **0** | **1** | **1** | **1** | **0** |
| **4** | **Kaya** | **2002** | **7** | **0** | **1** | **1** | **1** | **1** | **1** | **0** | **1** | **1** |
| **5** | **Kontula** | **1997** | **6** | **0** | **1** | **1** | **1** | **1** | **0** | **0** | **1** | **1** |
| **6** | **Lee** | **2002** | **8** | **1** | **1** | **1** | **1** | **1** | **1** | **1** | **1** | **0** |
| **7** | **Liu** | **2017** | **8** | **1** | **1** | **1** | **1** | **1** | **1** | **0** | **1** | **1** |
| **8** | **Mee** | **1998** | **6** | **1** | **1** | **0** | **1** | **1** | **1** | **0** | **1** | **0** |
| **9** | **Okita** | **2002** | **8** | **1** | **1** | **1** | **1** | **1** | **1** | **0** | **1** | **1** |
| **10** | **Park** | **1999** | **7** | **1** | **1** | **1** | **1** | **1** | **0** | **0** | **1** | **1** |
| **11** | **Richetta** | **2014** | **8** | **1** | **1** | **1** | **1** | **1** | **1** | **0** | **1** | **1** |
| **12** | **Richetta** | **2012** | **8** | **1** | **1** | **1** | **1** | **1** | **1** | **0** | **1** | **1** |
| **13** | **Ruggiero** | **2004** | **7** | **1** | **1** | **1** | **1** | **1** | **0** | **0** | **1** | **1** |
| **14** | **Saeki** | **2002** | **7** | **1** | **1** | **1** | **1** | **1** | **0** | **0** | **1** | **1** |
| **15** | **Zhao** | **2015** | **8** | **1** | **1** | **1** | **1** | **1** | **1** | **0** | **1** | **1** |
| **16** | **Zhou** | **2014** | **6** | **1** | **1** | **0** | **0** | **1** | **1** | **0** | **1** | **1** |
| **17** | **Zhu** | **2002** | **7** | **0** | **1** | **1** | **1** | **1** | **1** | **0** | **1** | **1** |
| **18** | **Zuel** | **2011** | **6** | **0** | **1** | **1** | **1** | **1** | **0** | **0** | **1** | **1** |

Note: *NOS* Newcastle-Ottawa quality assessment scale,

*Term 1* Is ths case definition adequate?-with independent validation,

*Term 2* Representativeness of cases-consecutive or obviously representative series of cases,

*Term 3* Selection of controls-community controls,

*Term 4* Definition of controls-no history of disease,

*Term 5* Comparability of cases and controls on the basis of the design or analysis-study controls for the most important factor,

*Term 6* Comparability of cases and controls on the basis of the design or analysis-study controls for any additional factor,

*Term 7* secure record of exposure-secure record,

*Term 8* same method of ascertainment for cases and controls,

*Term 9* Non-response rate-same rate for both groups
