# Supplementary figures and images for: Pooling analysis regarding the impact of human vitamin D receptor variants on the odds of psoriasis
Source: BMC Med Genet. 2019 Oct 17;20:161. doi: 10.1186/s12881-019-0896-6 (PMC6796361; doi:10.1186/s12881-019-0896-6)

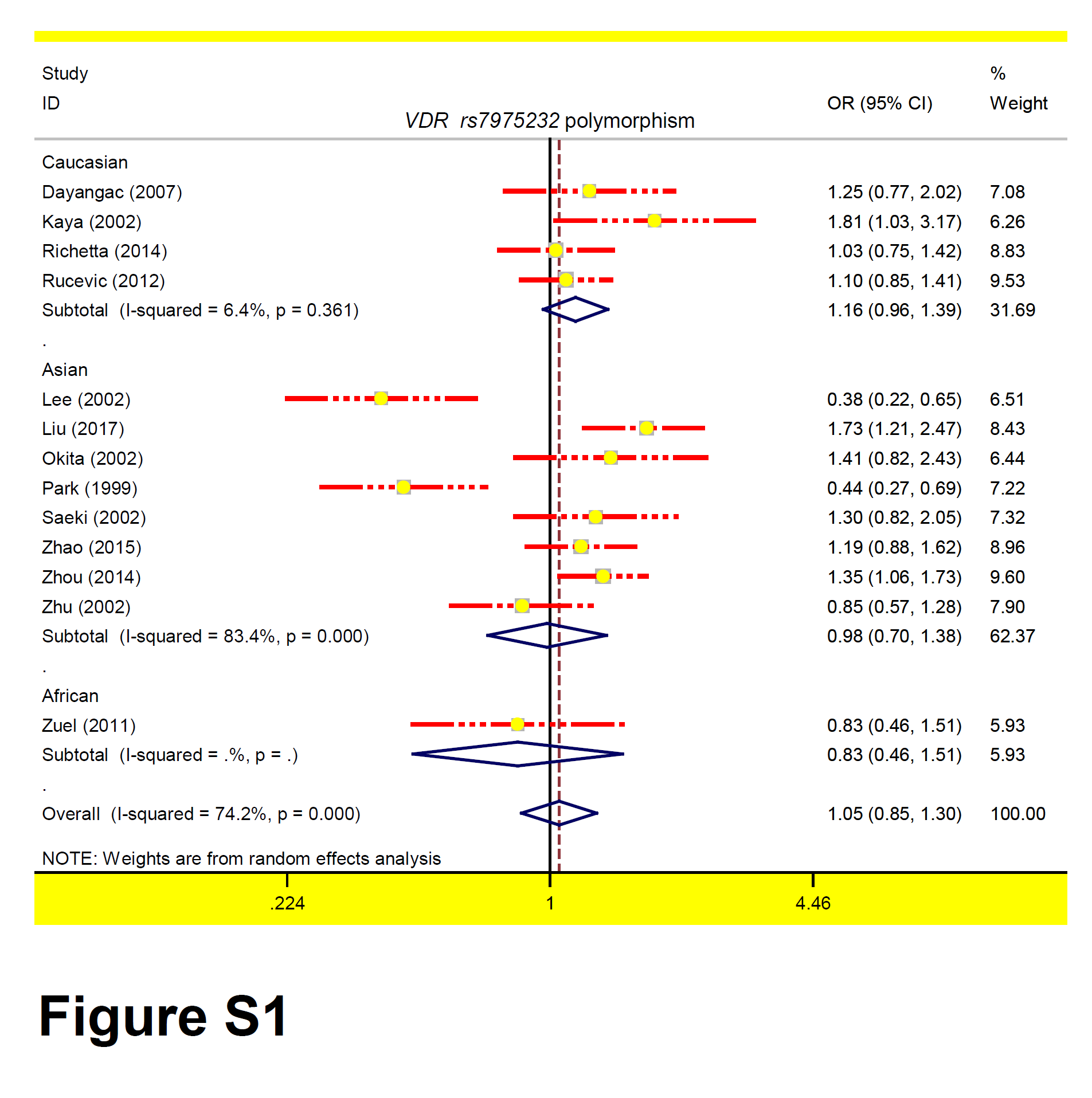

Supplement: Supplementary file 4 — Additional file 4: Figure S1. The forest plot for VDR rs7975232 polymorphism in the subgroup analysis by ethnicity under the allele model. [file 12881_2019_896_MOESM4_ESM.tif]

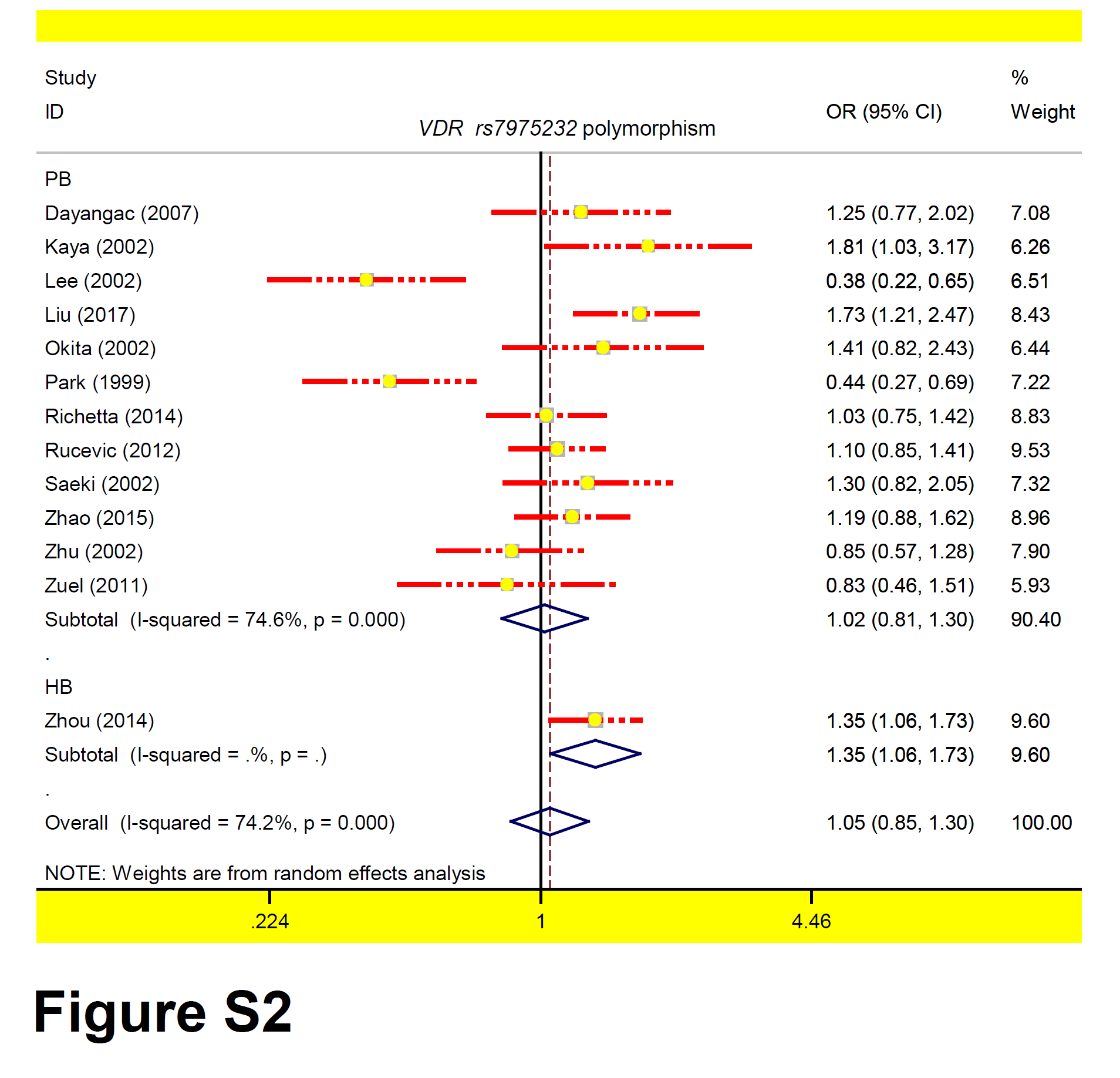

Supplement: Supplementary file 5 — Additional file 5: Figure S2. The forest plot for VDR rs7975232 polymorphism in the subgroup analysis by the source of controls under the allele model. [file 12881_2019_896_MOESM5_ESM.tif]

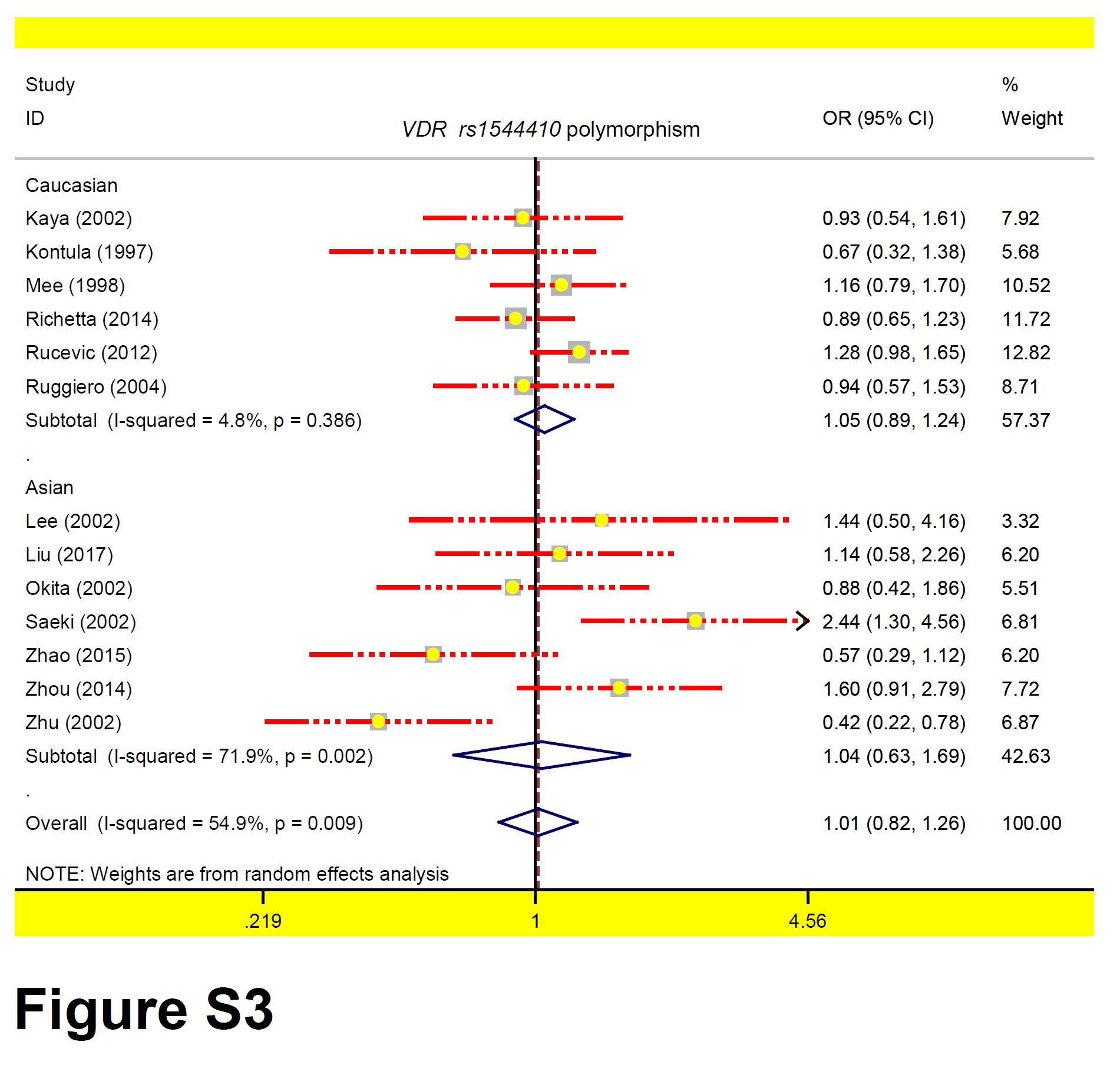

Supplement: Supplementary file 6 — Additional file 6: Figure S3. The forest plot for VDR rs1544410 polymorphism in the subgroup analysis by ethnicity under the allele model. [file 12881_2019_896_MOESM6_ESM.tif]

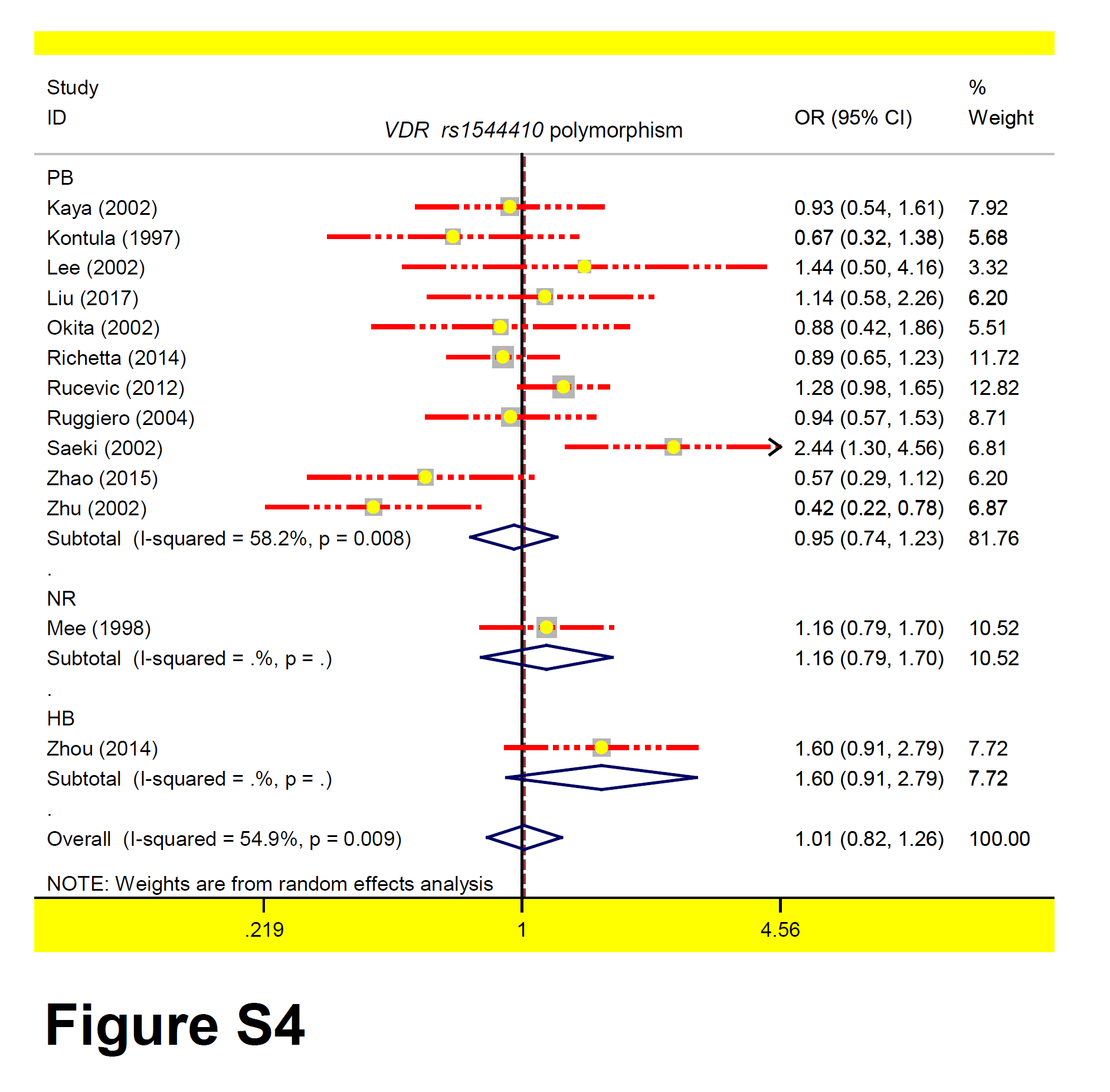

Supplement: Supplementary file 7 — Additional file 7: Figure S4. The forest plot for VDR rs1544410 polymorphism in the subgroup analysis by the source of controls under the allele model. [file 12881_2019_896_MOESM7_ESM.tif]

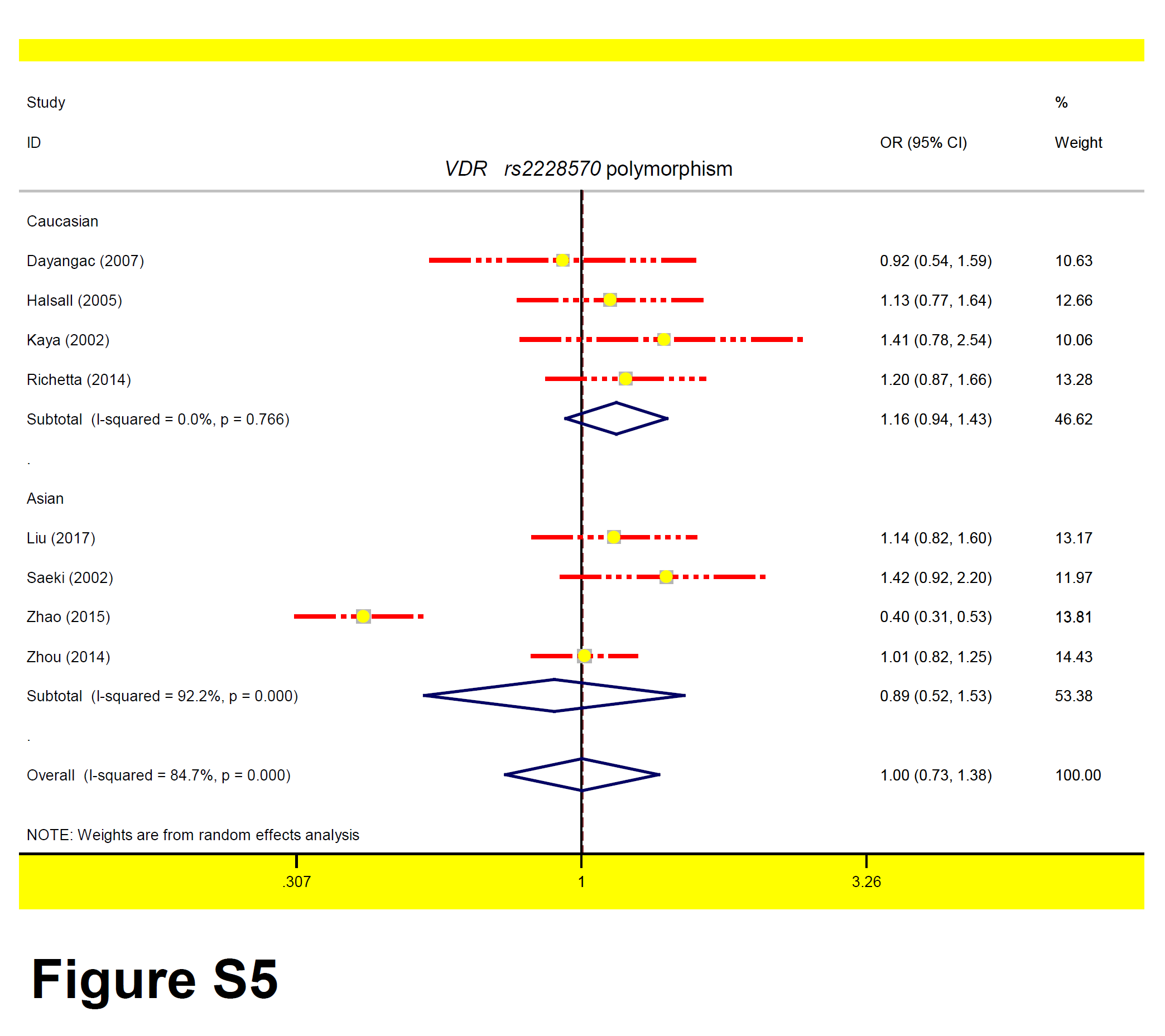

Supplement: Supplementary file 8 — Additional file 8: Figure S5. The forest plot for VDR rs2228570 polymorphism in the subgroup analysis by ethnicity under the allele model. [file 12881_2019_896_MOESM8_ESM.tif]

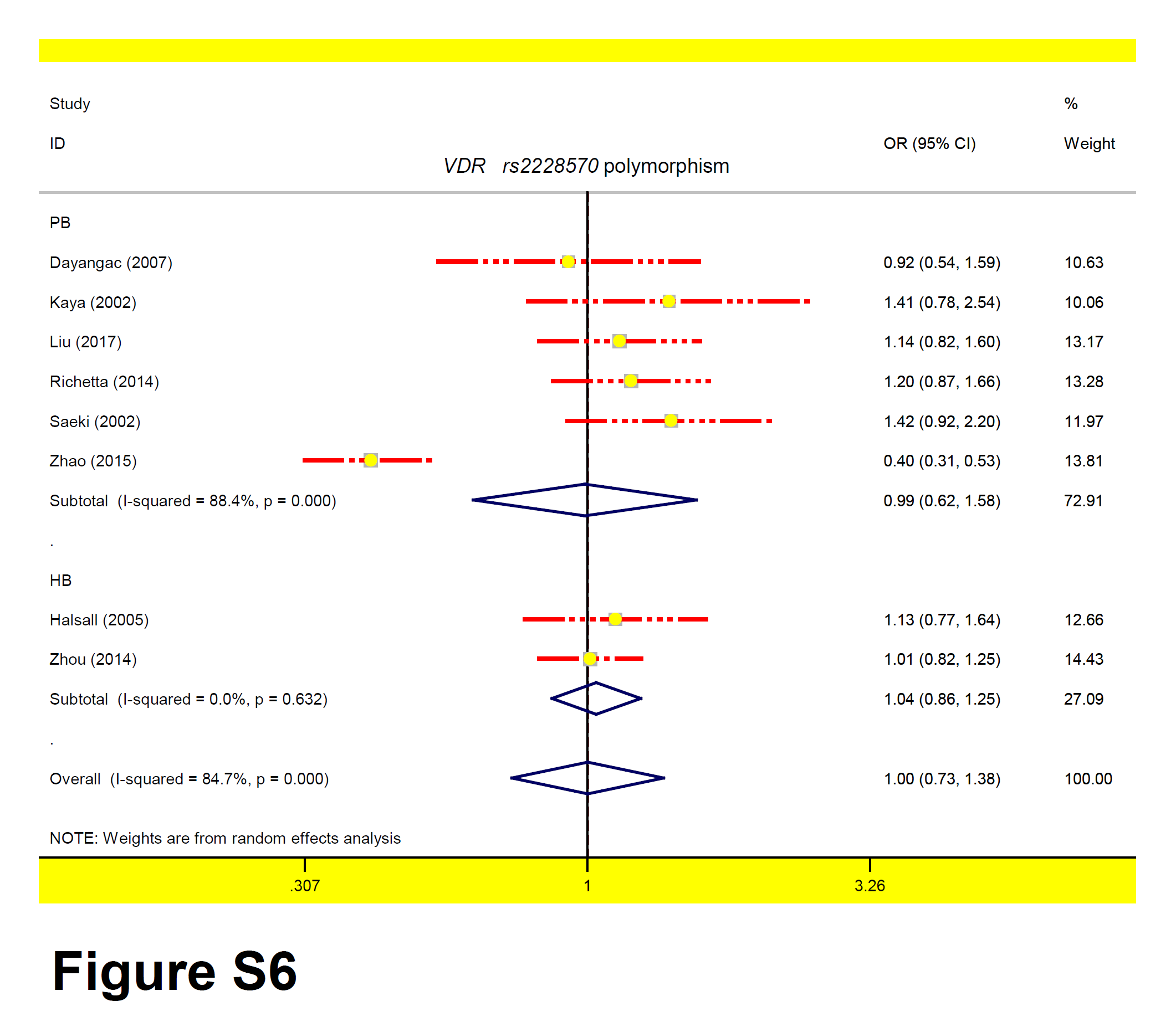

Supplement: Supplementary file 9 — Additional file 9: Figure S6. The forest plot for VDR rs2228570 polymorphism in the subgroup analysis by the source of controls under the allele model. [file 12881_2019_896_MOESM9_ESM.tif]

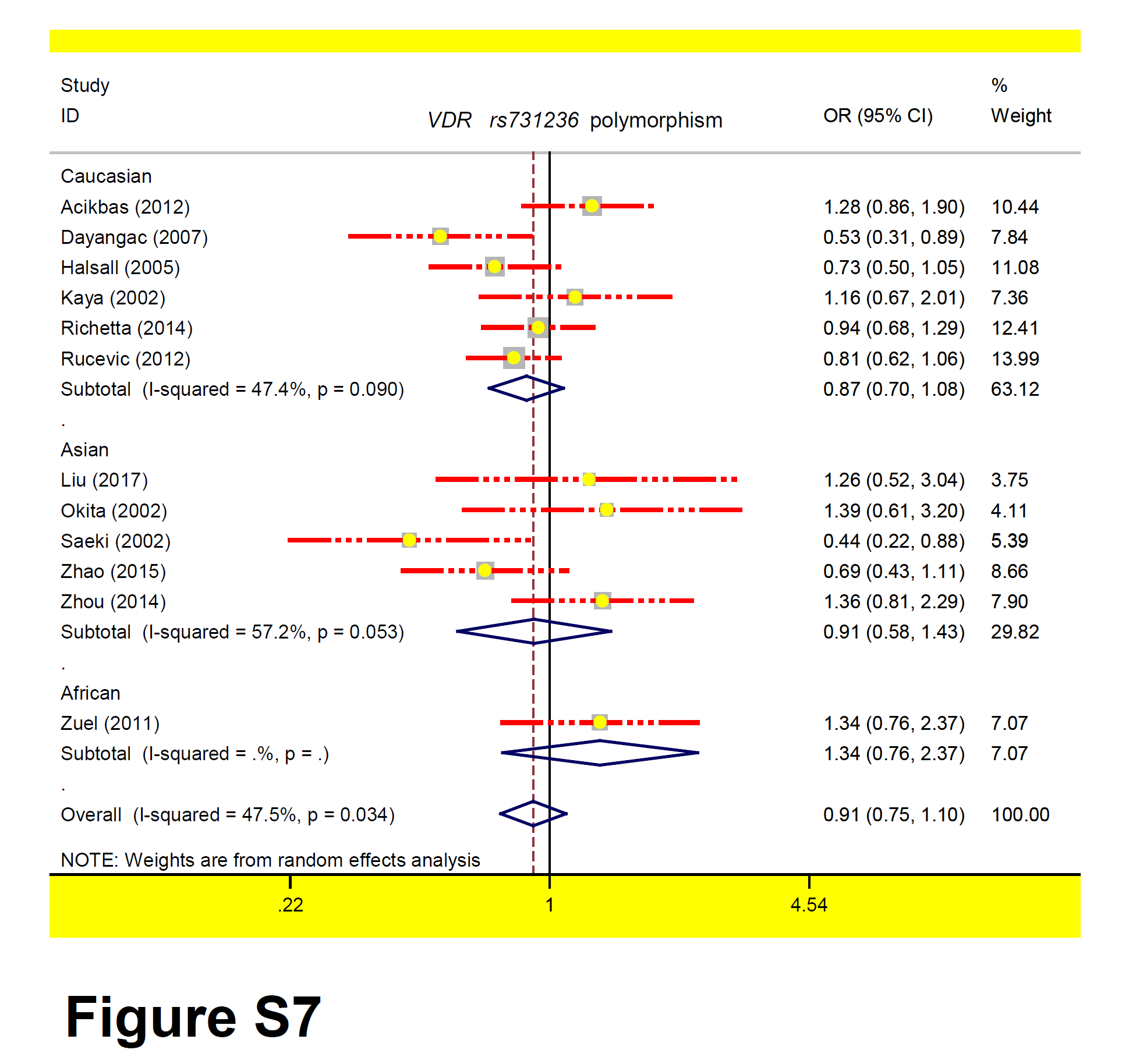

Supplement: Supplementary file 10 — Additional file 10: Figure S7. The forest plot for VDR rs731236 polymorphism in the subgroup analysis by ethnicity under the allele model. [file 12881_2019_896_MOESM10_ESM.tif]

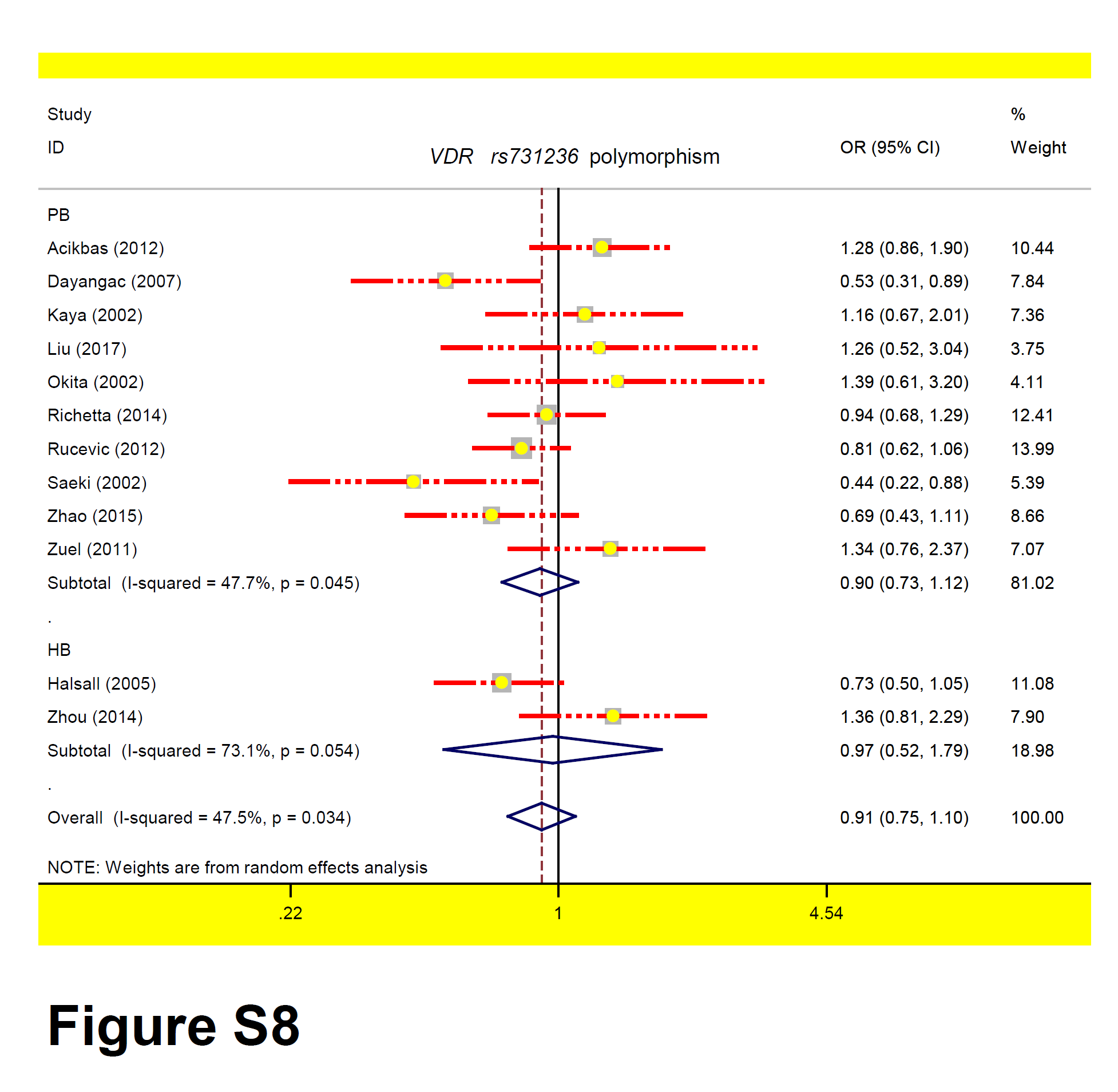

Supplement: Supplementary file 11 — Additional file 11: Figure S8. The forest plot for VDR rs731236 polymorphism in the subgroup analysis by the source of controls under the allele model. [file 12881_2019_896_MOESM11_ESM.tif]

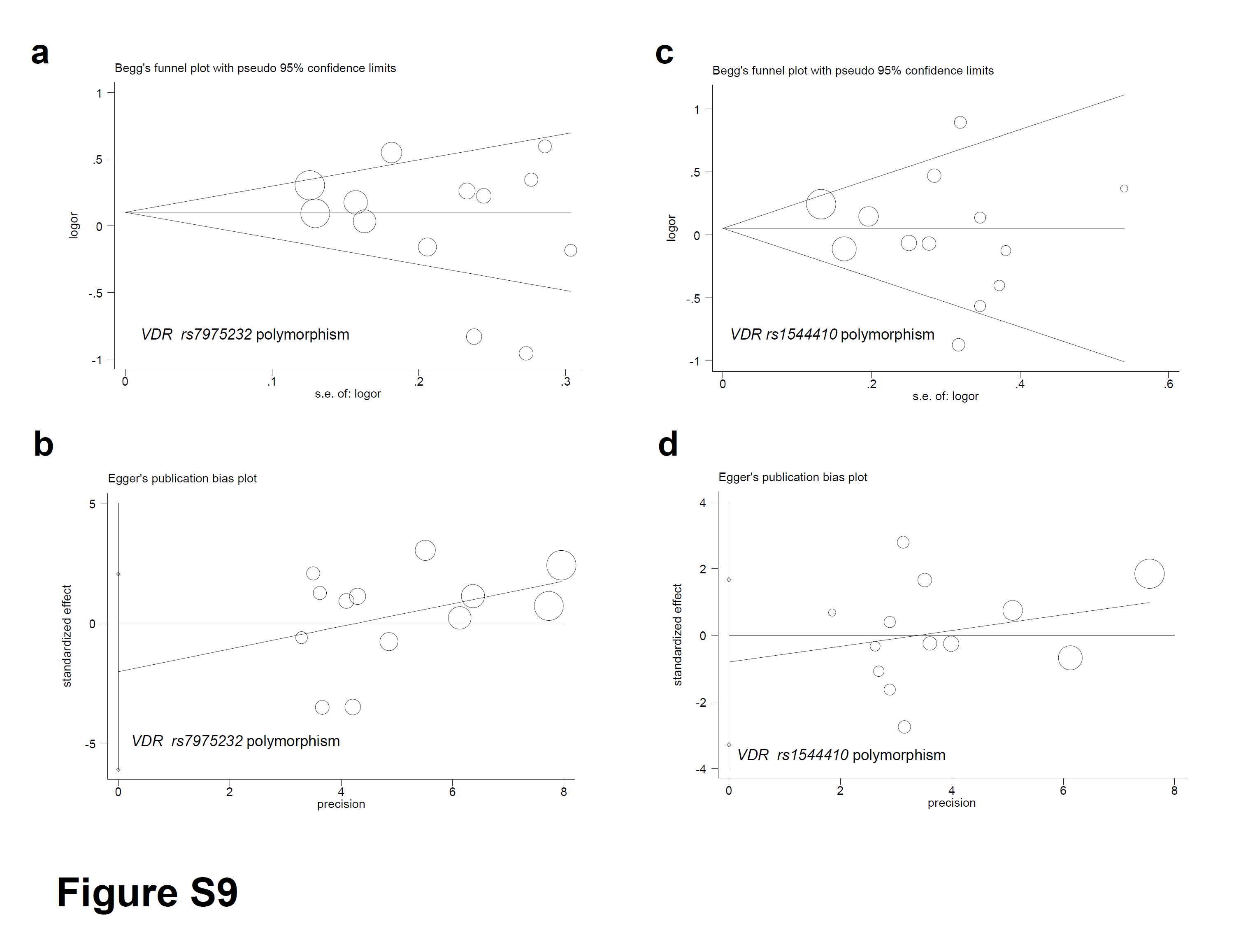

Supplement: Supplementary file 12 — Additional file 12: Figure S9. Publication bias of VDR rs7975232 and rs1544410 polymorphism under the allele model. a-b rs7975232 polymorphism; c-d rs1544410 polymorphism. [file 12881_2019_896_MOESM12_ESM.tif]

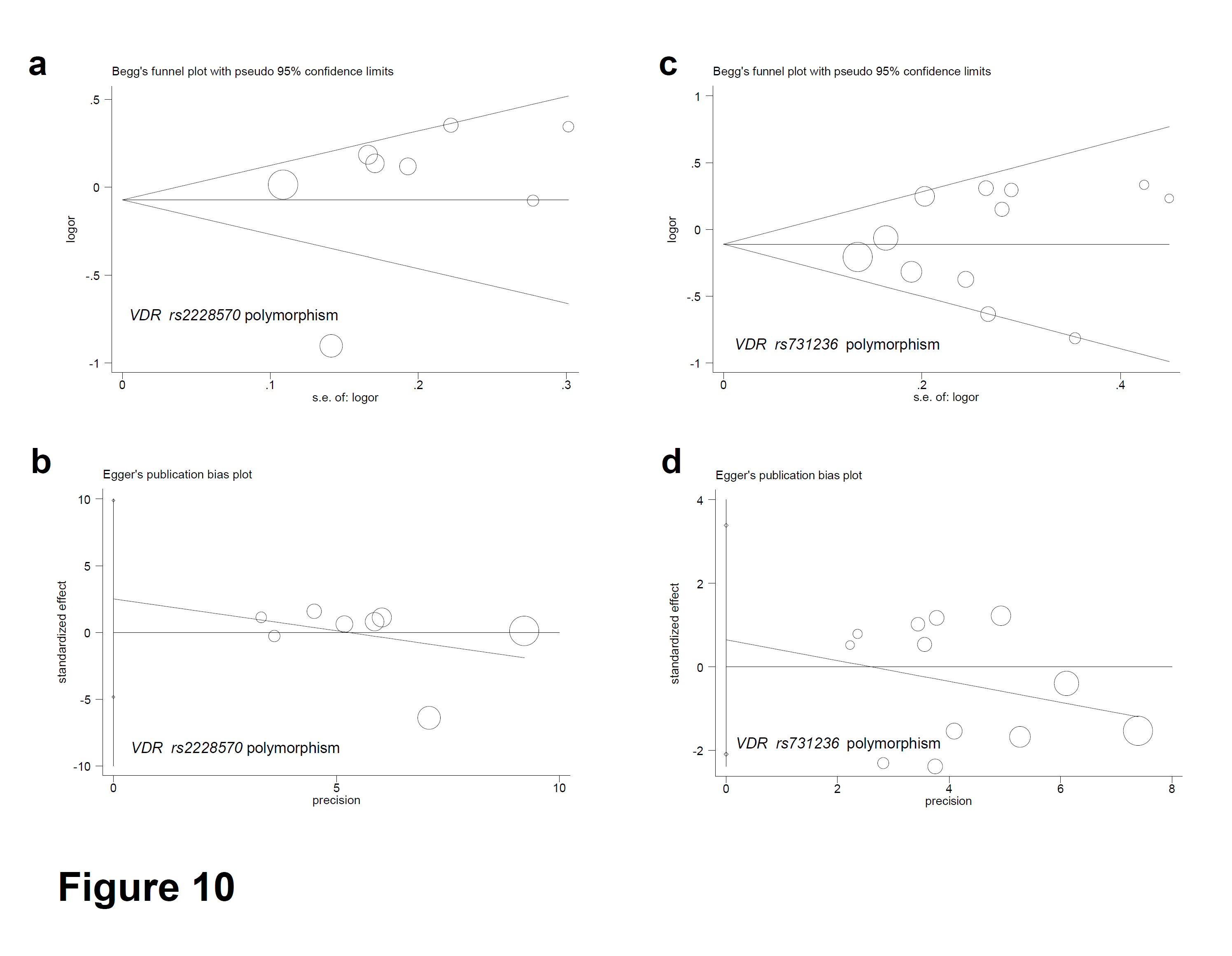

Supplement: Supplementary file 13 — Additional file 13: Figure S10. Publication bias of VDR rs2228570 and rs731236 polymorphism under the allele model. a-b rs2228570 polymorphism; c-d rs731236 polymorphism. [file 12881_2019_896_MOESM13_ESM.tif]
